# TIAM1-mediated synaptic plasticity underlies comorbid depression–like and ketamine antidepressant–like actions in chronic pain

**DOI:** 10.1172/JCI158545

**Published:** 2022-12-15

**Authors:** Qin Ru, Yungang Lu, Ali Bin Saifullah, Francisco A. Blanco, Changqun Yao, Juan P. Cata, De-Pei Li, Kimberley F. Tolias, Lingyong Li

**Affiliations:** 1 Department of Neuroscience, Baylor College of Medicine, Houston, Texas, USA.; 2 Department of Health and Kinesiology, School of Physical Education, Jianghan University, Wuhan, China.; 3 Integrative Molecular and Biomedical Sciences Graduate Program, Baylor College of Medicine, Houston, Texas, USA.; 4 Department of Anesthesiology and Perioperative Medicine, The University of Texas, MD Anderson Cancer Center, Houston, Texas, USA.; 5 Center for Precision Medicine, Department of Medicine, School of Medicine, University of Missouri, Columbia, Missouri, USA.; 6 Verna and Marrs McLean Department of Biochemistry and Molecular Biology, Baylor College of Medicine, Houston, Texas, USA.; 7 Division of Molecular and Translational Biomedicine, Department of Anesthesiology and Perioperative Medicine, Heersink School of Medicine, University of Alabama at Birmingham, Birmingham, Alabama, USA.

**Keywords:** Neuroscience, Depression, Neurological disorders, Pain

## Abstract

Chronic pain often leads to depression, increasing patient suffering and worsening prognosis. While hyperactivity of the anterior cingulate cortex (ACC) appears to be critically involved, the molecular mechanisms underlying comorbid depressive symptoms in chronic pain remain elusive. T cell lymphoma invasion and metastasis 1 (Tiam1) is a Rac1 guanine nucleotide exchange factor (GEF) that promotes dendrite, spine, and synapse development during brain development. Here, we show that Tiam1 orchestrates synaptic structural and functional plasticity in ACC neurons via actin cytoskeleton reorganization and synaptic *N*-methyl-d-aspartate receptor (NMDAR) stabilization. This Tiam1-coordinated synaptic plasticity underpins ACC hyperactivity and drives chronic pain–induced depressive-like behaviors. Notably, administration of low-dose ketamine, an NMDAR antagonist emerging as a promising treatment for chronic pain and depression, induces sustained antidepressant-like effects in mouse models of chronic pain by blocking Tiam1-mediated maladaptive synaptic plasticity in ACC neurons. Our results reveal Tiam1 as a critical factor in the pathophysiology of chronic pain–induced depressive-like behaviors and the sustained antidepressant-like effects of ketamine.

## Graphical abstract

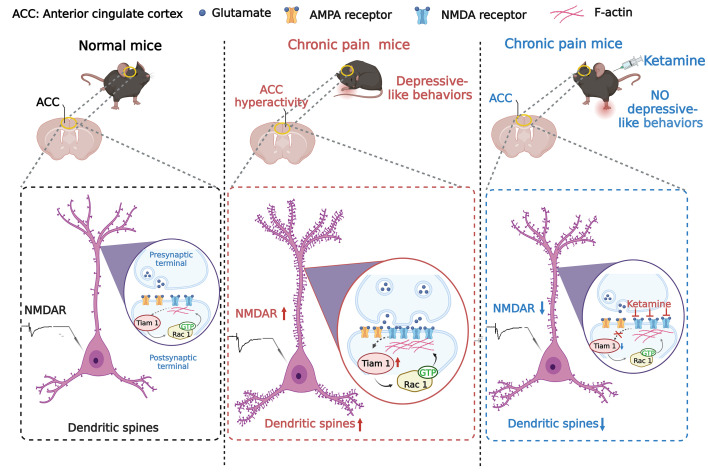

## Introduction

Chronic pain and depression are frequently comorbid, and their coexistence tends to worsen the severity of both disorders (1–4). Clinical and preclinical studies have established that anterior cingulate cortex (ACC) hyperactivity is essential for driving the comorbid depressive symptoms in chronic pain (5–8). Indeed, optogenetic activation of pyramidal neurons within the ACC is sufficient to induce depressive-like behaviors in naive mice (5), whereas lesioning the ACC or optogenetic inhibition of pyramidal neuron hyperactivity within the ACC blocks chronic pain–induced depressive-like behaviors without affecting sensory mechanical sensitivity (5–7, 9). While these findings argue that ACC hyperactivity underlies the comorbid depressive behaviors in chronic pain, the cause of ACC hyperactivity remains unknown. Notably, the noncompetitive *N*-methyl-d-aspartate receptor (NMDAR) antagonist ketamine has emerged as an effective treatment for both pain and comorbid depressive symptoms. Low, subanalgesic doses of ketamine can produce rapid, long-lasting antidepressant-like effects in patients and animal models (10, 11). However, the underlying mechanisms of ketamine’s antidepressant effects have not been fully elucidated.

Rho GTPases (e.g., Rac1, RhoA) play essential roles in dendritic spine morphogenesis and synaptic plasticity by controlling the organization of the underlying actin cytoskeleton (12). Precise spatiotemporal regulation of Rho GTPases is mediated by guanine nucleotide exchange factors (GEFs), which activate Rho GTPases by facilitating GDP/GTP exchange, and GTPase-activating proteins (GAPs), which inhibit Rho GTPases by stimulating GTP hydrolysis. We previously identified the Rac1 GEF T cell lymphoma invasion and metastasis 1 (Tiam1) as a critical regulator of dendritic spine and synapse development that couples synaptic NMDAR activity to Rac1 activation and actin cytoskeletal remodeling in developing hippocampal neurons (13–15). Recently, Tiam1 has also been shown to form a reciprocally activating signaling complex (RAKEC) with CaMKIIα, which stably activates CaMKIIα and Rac1 and maintains spine morphology during long-term potentiation (LTP) (16). Here, we establish Tiam1 as an essential factor in the pathophysiology of comorbid depression in chronic pain through its regulation of maladaptive synaptic plasticity in ACC neurons, which can be targeted by ketamine to produce sustained antidepressant-like effects.

## Results

### Tiam1 in the ACC is activated in mouse models of chronic pain with depressive/anxiety-like behaviors.

**Figure 1:**
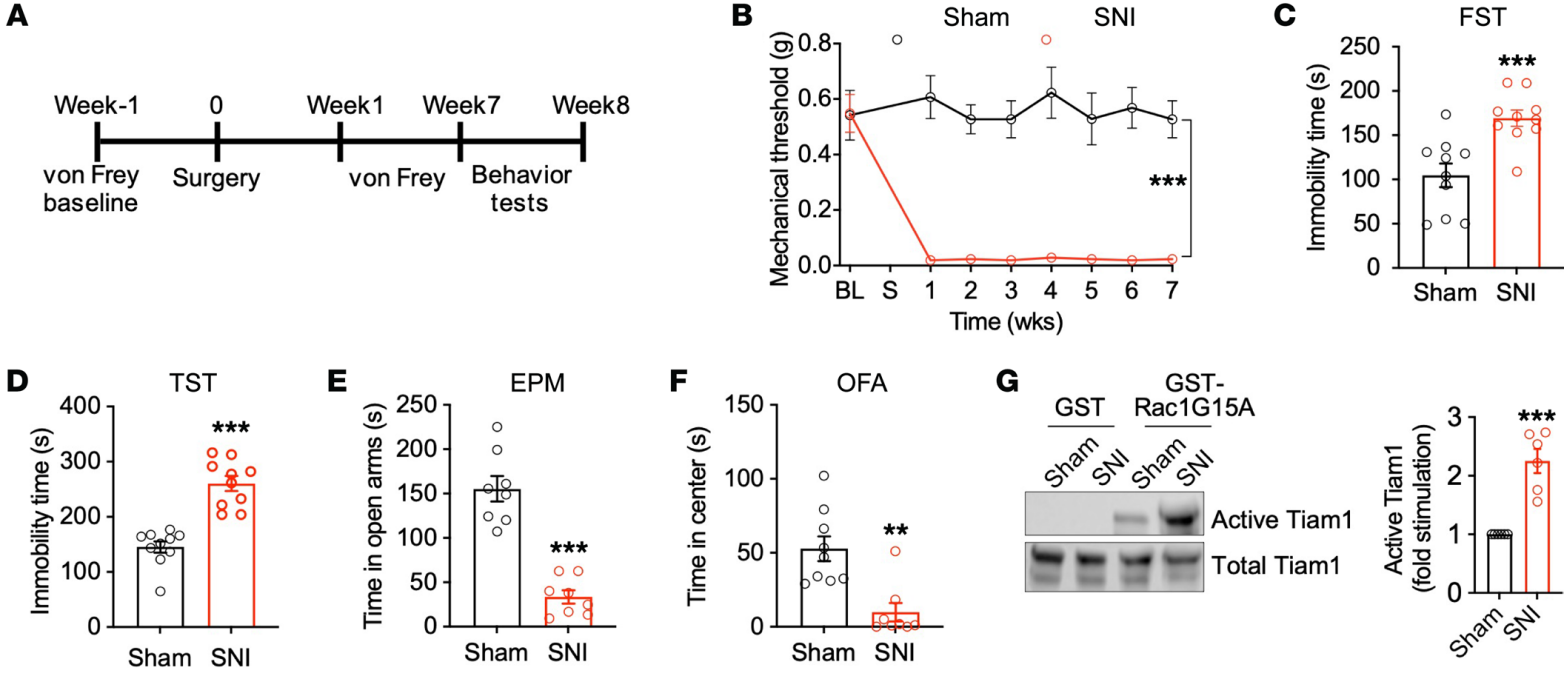
Tiam1 is activated in the ACC of mouse models of chronic pain displaying depressive/anxiety-like behaviors. (**A**) Experimental paradigm. (**B**) Time course of SNI-induced sensory pain (*n* = 7 mice for each group). BL, baseline; S, sham or SNI surgery. (**C**–**F**) Mice with neuropathic pain (7 weeks after SNI surgery) displayed depressive/anxiety-like behaviors, as shown by increased immobile times in the FST and TST and reductions in the duration in open arms in the EPM test and time in center in the OFA test. Sham, *n* = 9 mice; SNI, *n* = 8 mice. (**G**) Representative blot and quantification of an affinity-precipitation active GEF assay with GST (control) and GST-Rac1G15A (nucleotide-free Rac1) showing Tiam1 activation in the ACC of mice 7 weeks after sham treatment or SNI (*n* = 6 mice). Data are represented as mean ± SEM. **P < 0.01; ***P < 0.001. Two-way ANOVA followed by Tukey’s post hoc test (**B**); 2-tailed unpaired Student’s *t* test (**C**–**G**).

The ACC appears to be a critical hub for comorbid depressive symptoms in chronic pain (5, 8, 9). NMDAR-mediated enhancement of excitatory synaptic transmission in the ACC coincides with comorbid depressive–like behaviors in neuropathic pain (9). Based on our previous finding that Tiam1 couples synaptic NMDARs to Rac1-dependent actin cytoskeletal reorganization and dendritic spine and synapse development in hippocampal neurons (13–15), we explored the possible role of Tiam1-mediated synaptic remodeling in ACC hyperactivity and chronic pain–induced depressive-like behaviors. Chronic pain is broadly classified as neuropathic pain or inflammatory pain caused by nerve or tissue damage, respectively, stemming from various injuries (17). To investigate Tiam1’s role in chronic pain–induced depressive-like behaviors, we used the spared nerve injury–induced (SNI-induced) neuropathic pain model and the CFA-induced inflammatory pain model (Figure 1, A and B, and Supplemental Figure 1, A and B; supplemental material available online with this article; https://doi.org/10.1172/JCI158545DS1), which are both well-accepted mouse models of chronic pain (18, 19). As expected, 7 weeks after SNI surgery, WT mice displayed depressive- and anxiety-like behaviors in several established behavioral assays, including the forced swim test (FST) (increased immobility), the tail-suspension test (TST) (increased immobility), the elevated-plus-maze (EPM) test (reduced open-arm time), and the open-field activity (OFA) test (reduced center-zone time) (Figure 1, C–F). Similar depressive/anxiety-like behaviors were observed in WT mice 3 weeks after CFA injection (Supplemental Figure 1, C and D), confirming that depressive/anxiety-like behaviors are reliably induced by these chronic pain models.

To investigate whether Tiam1 is activated in the ACC of mouse models of chronic pain displaying depressive/anxiety-like behaviors, we carried out an affinity-precipitation assay to detect active Tiam1 using GST-Rac1G15A, a guanine nucleotide–free form of Rac1 that preferentially binds to activated GEFs (20). Seven weeks after sham or SNI surgery or 3 weeks after saline or CFA injection, the active GEF assay was performed on ACC homogenates from the mice. We found that the levels of Tiam1 that precipitated with Rac1G15A from SNI mice were markedly increased compared with those of sham-treated controls (Figure 1G). Furthermore, Tiam1 was activated in a time-dependent manner after nerve injury, reaching maximum activation 8 weeks after SNI that was sustained at 24 weeks (Supplemental Figure 2). However, we did not detect significant Tiam1 activation after SNI surgery in other brain regions that are thought to be involved in the comorbidity between pain and mood disorders, including the amygdala, nucleus accumbens (NAc), insular cortex, or prefrontal cortex (PFC) (21) (Supplemental Figure 3). Like SNI, Tiam1 activation was detected in the ACC of CFA mice in contrast with saline controls (Supplemental Figure 4). These data indicate that ACC Tiam1 is activated in mouse models of chronic pain with depressive/anxiety-like behaviors.

### Tiam1 in the ACC modulates chronic pain–induced depressive-like behaviors.

To investigate the causal link between ACC Tiam1 activation and depressive/anxiety-like behaviors, we utilized *Tiam1*-floxed mice (*Tiam1^fl/fl^*) previously generated in our lab (15). To delete Tiam1 from postnatal forebrain excitatory neurons, we crossed *Tiam1*-floxed mice with a CaMKIIα-Cre transgenic line. We confirmed Tiam1 loss in the forebrain of these *Tiam1*–conditional KO (cKO) mice via Western blot analysis (Supplemental Figure 5A). To further verify the selective deletion of Tiam1 from CaMKIIα-expressing neurons in *Tiam1*-cKO mice, we injected Cre-dependent AAV vector expressing EGFP (AAV8-pCAG-DIO-EGFP) into the ACC of *Tiam1*-cKO mice (CaMKIIα-Cre:*Tiam1^fl/fl^*) and control mice (CaMKIIα-Cre) and isolated EGFP-expressing ACC neurons with flow cytometry. Western blot analysis revealed that Tiam1 was selectively deleted from CaMKIIα-expressing neurons in *Tiam1*-cKO mice (Supplemental Figure 5B). Mice lacking Tiam1 are viable, fertile, and display no gross alterations in the spinal cord or brain structures (15). *Tiam1*-cKO mice also performed as well as littermate controls on the rotarod (Supplemental Figure 5C), suggesting that they do not have deficits in motor coordination, motor learning, or balance.

**Figure 2:**
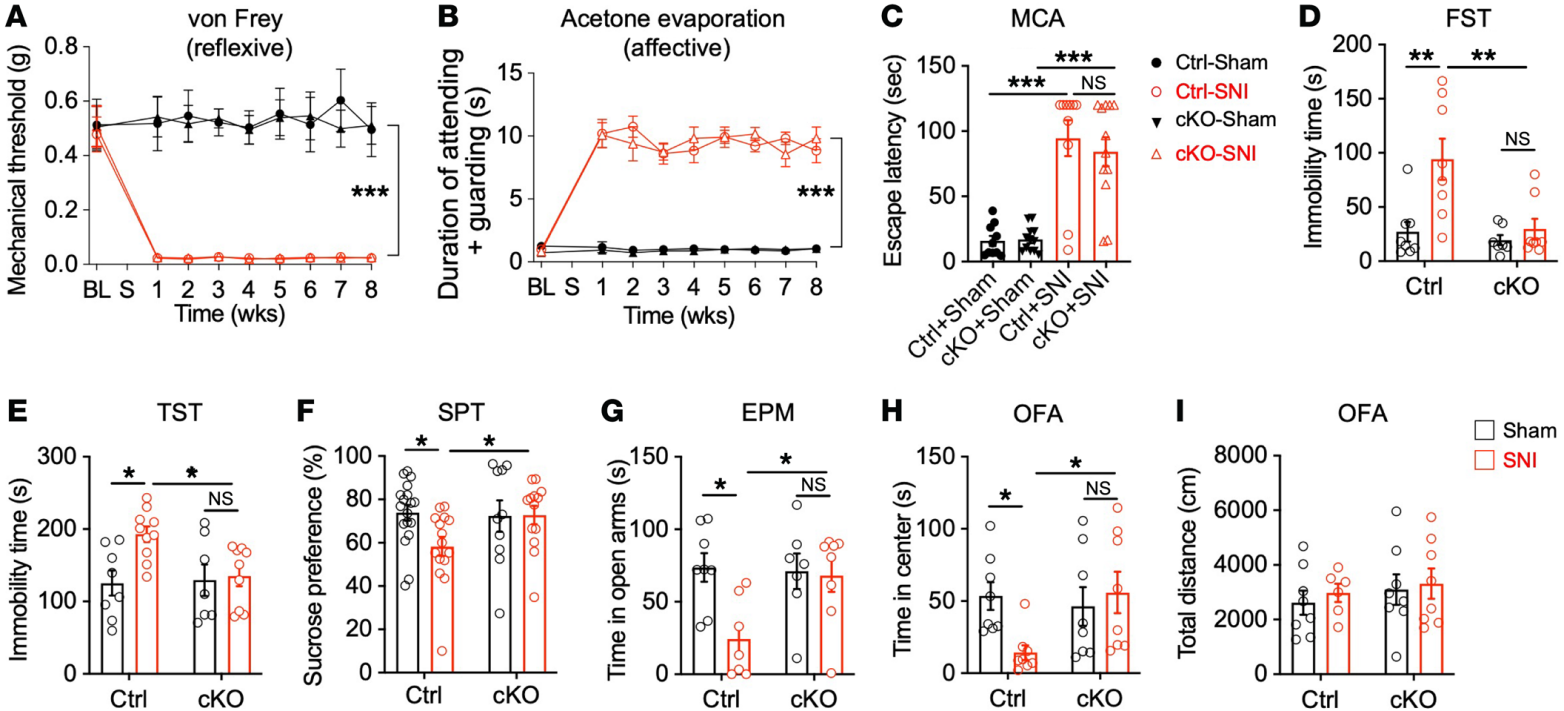
Conditional deletion of Tiam1 from postnatal forebrain excitatory neurons prevents chronic pain–induced depressive/anxiety-like behaviors. (**A** and **B**) Time course of SNI-induced pain reflexive behavior, demonstrated by a von Frey mechanical threshold assay (**A**, control-sham [Ctrl-sham], *n* = 5 mice; Ctrl-SNI, *n* = 5 mice; cKO-sham, *n* = 7 mice; cKO-SNI, *n* = 7 mice) and pain affective-motivational behavior in response to acetone evaporation (**B**, *n* = 9 mice for each group) in control and *Tiam1*-cKO mice before and during the 8 weeks following surgery. (**C**) Escape latency from white-lit chamber in the MCA with 5 mm probe height in the conflict chamber 7 weeks after sham or SNI surgery (Ctrl-sham, *n* = 10 mice; Ctrl-SNI, *n* = 10 mice; cKO-sham, *n* = 12 mice; cKO-SNI, *n* = 12 mice). (**D**–**H**) Behavioral tests demonstrating that chronic neuropathic pain (7 weeks after SNI surgery) induced depressive/anxiety-like behaviors in control mice, but not in *Tiam1*-cKO mice, in the FST (**D**), TST (**E**), SPT (**F**) and EPM (**G**) and OFA (**H**) tests (Ctrl-sham, *n* = 8 mice; Ctrl-SNI, *n* = 8 mice; cKO-sham, *n* = 7 mice; cKO-SNI, *n* = 8 mice; FST). (**I**) OFA showing that chronic neuropathic pain had no effect on locomotion in control or *Tiam1*-cKO mice (*n* = 7-8 mice). Data are represented as mean ± SEM. *P < 0.05; **P < 0.01; ***P < 0.001. Two-way ANOVA followed by Tukey’s post hoc test (**A**, **B**, **D**–**I**); 1-way ANOVA followed by Tukey’s post hoc test (**C**).

Chronic pain is a complex sensory and affective experience (22). To characterize chronic pain responses in *Tiam1*-cKO mice and littermate controls subjected to sham or SNI surgery, we tested mice for both reflexive withdraw via von Frey stimuli and affective-motivational behavior in response to acetone evaporation. Notably, we did not observe any difference in the mechanical withdrawal thresholds or attending and escape behaviors in control and *Tiam1*-cKO mice before or during the 8 weeks following SNI surgery (Figure 2, A and B). To further evaluate the effects of Tiam1 loss from postnatal forebrain excitatory neurons on chronic pain behaviors, we used an operant mechanical conflict-avoidance (MCA) test to measure nonreflexive pain behavior, which involves mice choosing to either remain in a brightly lit compartment or escape to a dark compartment by crossing an array of height-adjustable nociceptive probes (23, 24). Seven weeks after SNI surgery, both control and *Tiam1*-cKO mice exhibited a significant increase in escape latency from the white-lit chamber at a probe height of 5 mm, and there was no significant difference between them (Figure 2C), suggesting that Tiam1 deletion from postnatal forebrain excitatory neurons does not affect peripheral nerve injury–induced pain hypersensitivity in reflexive and affective dimensions.

Since *Tiam1*-cKO mice develop chronic pain similar to that of control littermates, we next explored whether Tiam1 plays a role in chronic pain–induced depressive/anxiety-like behaviors by testing control and *Tiam1*-cKO mice 7 weeks after sham or SNI surgery. Strikingly, we found that, in contrast with SNI-treated control mice, *Tiam1*-cKO mice subjected to SNI did not display depressive- or anxiety-like behaviors in the FST, the TST, the sucrose-preference test (SPT), the EPM test, and the OFA test and instead performed similarly to sham-treated animals (Figure 2, D–H). Likewise, in the CFA-induced inflammatory pain model, *Tiam1*-cKO mice displayed a marked reduction in depressive/anxiety-like behaviors compared with control mice (Supplemental Figure 6, A–C). Neither *Tiam1*-cKO nor control mice exhibited changes in locomotion in the OFA test in response to chronic pain (Figure 2I and Supplemental Figure 6D). Together, these results suggest that Tiam1 is essential in postnatal forebrain excitatory neurons for the development of chronic pain–induced depressive/anxiety-like behaviors. Given that *Tiam1*-cKO mice displayed a marked reduction in depressive/anxiety-like behaviors following neuropathic pain and inflammatory pain relative to that of control mice (Figure 2 and Supplemental Figure 6) and ACC Tiam1 was activated in both pain models (Figure 1G and Supplemental Figure 4), we assume that they share similar underlying mechanisms. Because most cases of depression in chronic pain are associated with neuropathic pain, which is clinically difficult to treat (2, 25), the SNI-induced neuropathic pain model was employed in the following mechanistic experiments, as in the previous study (2).

**Figure 3:**
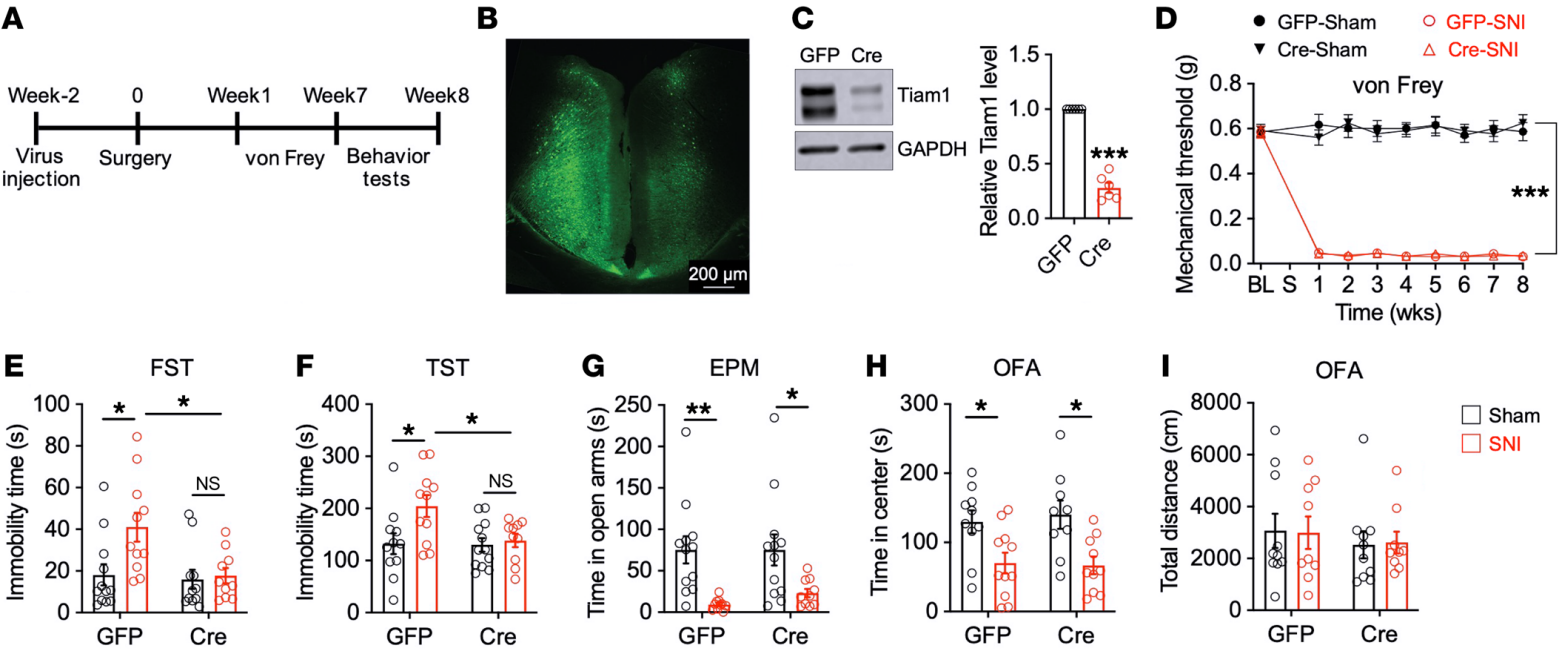
Tiam1 deletion from ACC neurons prevents chronic pain–induced depressive-like behaviors. (**A**) Experimental paradigm. (**B**) Representative image of the ACC after bilateral injections of rAAV8 (1 μl/site) expressing GFP. (**C**) Western blot analysis showing Cre-mediated Tiam1 loss in the ACC of *Tiam1*-floxed mice (*n* = 6 mice for each group). (**D**) Time course of pain hypersensitivity in response to von Frey filament stimuli in rAAV8-GFP– and rAAV8-Cre-GFP–injected *Tiam1*-floxed mice before and during the 8 weeks following sham or SNI surgery (GFP-sham, *n* = 12 mice; GFP-SNI, *n* = 11 mice; Cre-sham, *n* = 11 mice; Cre-SNI, *n* = 11 mice). (**E**–**I**) Behavioral tests demonstrating that Tiam1 deletion from ACC neurons prevented depressive-like behaviors in FST (**E**) and TST (**F**), but did not change anxiety-like behaviors in EPM (**G**) or OFA (**H**) tests or locomotor activity in OFA test (**I**) in mice with neuropathic pain (7 weeks after surgery) (GFP-sham, *n* = 12 mice; GFP-SNI, *n* = 11 mice; Cre-sham, *n* = 11 mice; Cre-SNI, *n* = 11 mice). Data are represented as mean ± SEM. *P < 0.05; **P < 0.01; ***P < 0.001. Two-tailed unpaired *t* test (**C**), 2-way ANOVA followed by Tukey’s post hoc test (**D**–**I**).

To further establish the functional role of ACC Tiam1 in chronic pain–induced depression/anxiety-like behaviors, we specifically deleted Tiam1 from ACC neurons of *Tiam1*-floxed mice by bilateral injection of an rAAV8 vector expressing Cre recombinase and GFP driven by the human synapsin 1 promoter (rAAV8-hSyn-Cre-GFP). An rAAV8 vector expressing GFP alone was used as the control (rAAV8-hSyn-GFP) (Figure 3, A–C, and Supplemental Figure 7). Specific deletion of Tiam1 from ACC neurons did not alter SNI-induced mechanical allodynia before or during the 8 weeks following surgery (Figure 3D), but it did significantly suppress depressive-like behaviors, as shown by a marked reduction in immobility times in the FST and TST (Figure 3, E and F). Interestingly, Tiam1 deletion from ACC neurons did not affect chronic pain–induced anxiety-like behaviors, as no significant change was observed in the open-arm time in the EPM test or center-zone time in the OFA test (Figure 3, G and H). As before, chronic pain did not induce locomotion changes in the OFA test in GFP- or Cre-injected *Tiam1*-floxed mice (Figure 3I). These data suggest that Tiam1 expressed in ACC neurons specifically mediates chronic pain–induced depressive-like behaviors.

### Tiam1 controls chronic pain–induced dendritic spine remodeling and functional synaptic plasticity in ACC neurons.

**Figure 4:**
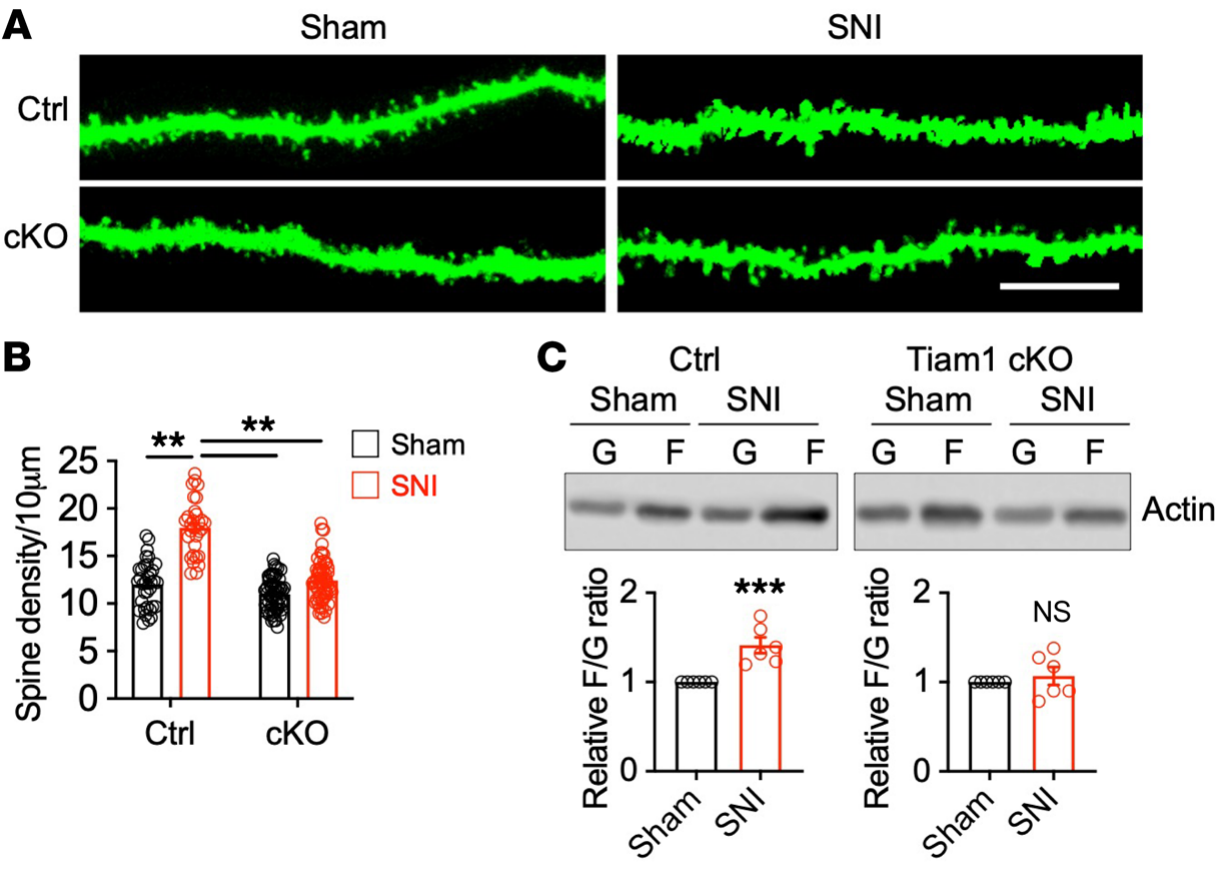
Tiam1 mediates synaptic structural plasticity of ACC neurons in mouse models of chronic pain. (**A** and **B**) Representative confocal images and summary of spine density analysis of the apical dendrites of ACC pyramidal neurons showing that chronic pain (7 weeks after SNI) increased the density of dendritic spines in control mice, but not in *Tiam1*-cKO mice (Ctrl-sham, *n* = 26 dendrites from 3 mice; Ctrl-SNI, *n* = 25 dendrites from 3 mice; cKO-sham, *n* = 30 dendrites from 3 mice; cKO-SNI, *n* = 30 dendrites from 3 mice). Scale bar: 10 μm. (**C**) Western blot and quantification analyses revealed that the ratio of F-actin (**F**) to G-actin (**G**) in the ACC was significantly increased in control mice, but not in *Tiam1*-cKO mice 7 weeks after SNI (*n* = 6 mice for each group). Data are represented as mean ± SEM. **P < 0.01; ***P < 0.001. Two-way ANOVA followed by Tukey’s post hoc test (**B**), 2-tailed unpaired *t* test (**C**).

Basic and clinical studies have established that an underlying cause of stress-induced depression and anxiety disorders is alterations in synaptic connections in brain regions involved in mood regulation, including the PFC, hippocampus, amygdala, and NAc (26, 27). Given that the ACC is a brain region that is important for processing the emotional features of pain (5–8), we investigated the possibility that chronic pain–induced depressive-like behaviors are caused by underlying synaptic alterations in the ACC. Dendritic spines are small actin-rich protrusions on dendrites that serve as the primary postsynaptic sites of excitatory synapses (28, 29). To probe for alterations in dendritic spines on ACC neurons following chronic pain, we injected a low titer of rAAV8-hSyn-GFP into the ACC of mice to sparsely label ACC neurons 2 weeks before surgery. We subjected these GFP-expressing mice to sham or SNI surgery and analyzed dendritic spines on secondary and tertiary dendrites of GFP-positive ACC neurons 7 weeks after surgery, a time when SNI mice display depressive-like behaviors. ACC neurons in control mice subjected to SNI showed significant increases in dendritic spine density compared with ACC neurons in sham-treated control mice (Figure 4, A and B), suggesting that chronic pain promotes dendritic spinogenesis in the ACC. Since actin polymerization is a driving force for dendritic spine remodeling, we next determined whether chronic pain modulates actin dynamics in the ACC. Actin exists in 2 forms: monomeric globular actin (G-actin) and polymerized filamentous actin (F-actin), which is composed of aggregated G-actin. The transition between these 2 forms of actin is controlled by synaptic activity (29). Using Western blot analysis to measure the levels of F- and G-actin, we found that the ratio of F- to G-actin, which reflects the balance between actin polymerization and depolymerization, was significantly increased in the ACC of control mice 7 weeks after SNI (Figure 4C). Together, these data indicate that chronic pain induces synaptic structural remodeling in ACC neurons.

**Figure 5:**
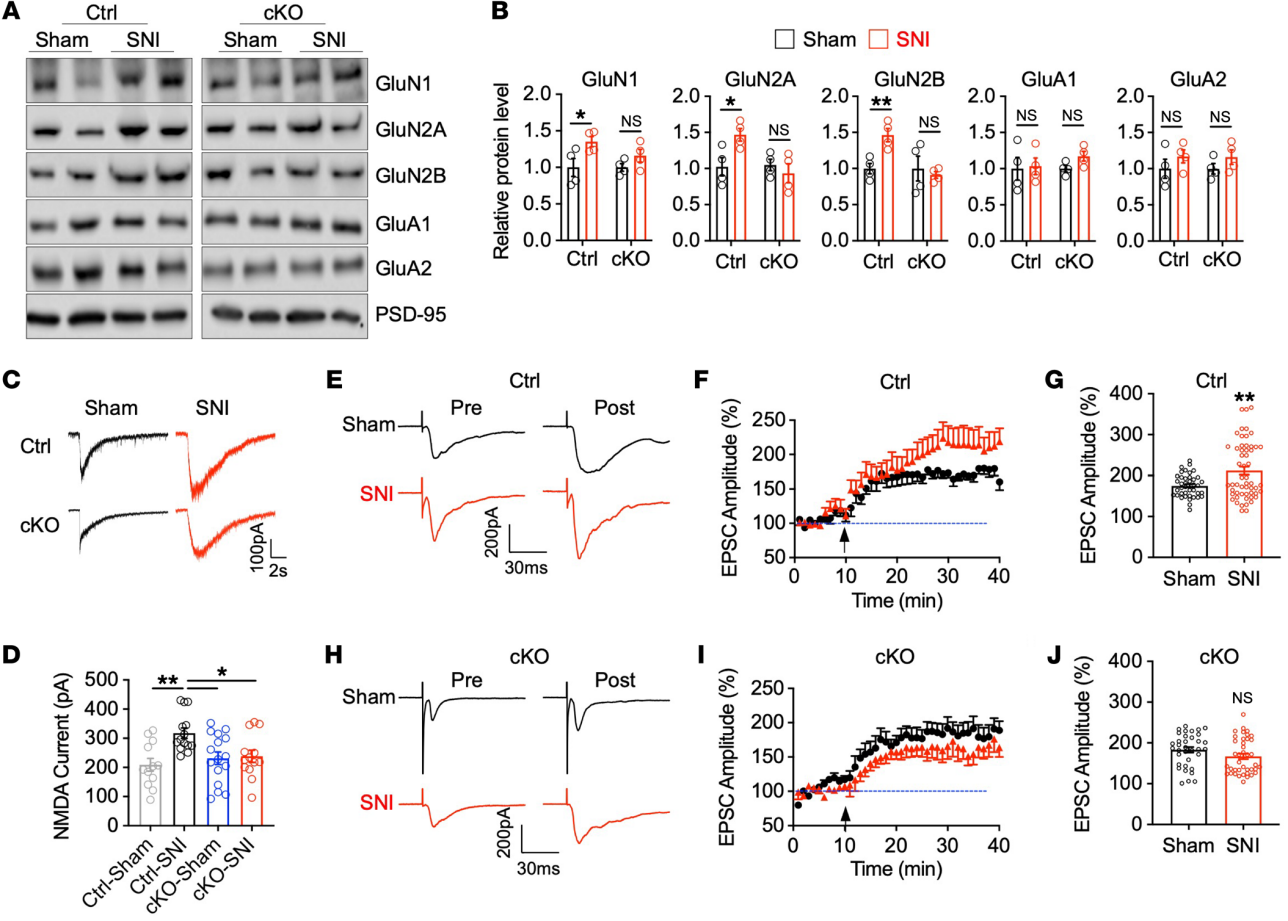
Tiam1 mediates synaptic functional plasticity of ACC neurons in mouse models of chronic pain. (**A** and **B**) Western blot and quantification analyses of synaptic NMDAR and AMPAR subunit protein levels in the ACC in control mice and *Tiam1*-cKO mice 7 weeks after sham or SNI surgery (*n* = 4 mice for each group). (**C** and **D**) Representative traces and mean changes in NMDAR currents elicited by puff application of 100 μM NMDA to ACC pyramidal neurons in control and *Tiam1*-cKO mice 7 weeks after sham or SNI surgery (Ctrl-sham, *n* = 12 neurons from 3 mice; Ctrl-SNI, *n* = 14 neurons from 4 mice; cKO-sham, *n* = 16 neurons from 4 mice; cKO-SNI, *n* = 13 neurons from 3 mice). (**E**-**J**) LTP was induced in pyramidal neurons in the ACC of control mice and *Tiam1*-cKO mice by the pairing training protocol. Averages of 6 EPSCs 5 minutes before and 25 minutes after the pairing training (arrows in **F** and **I**). (**E** and **H**), mean time courses (**F** and **I**), and summary of LTP induction (last 5 minutes, **G** and **J**) in ACC pyramidal neurons from control mice and *Tiam1*-cKO mice subjected to sham or SNI surgery (Ctrl-sham, *n* = 8 neurons from 6 mice; Ctrl-SNI, *n* = 11 neurons from 9 mice; cKO-sham, *n* = 10 neurons from 8 mice; cKO-SNI, *n* = 8 neurons from 6 mice). Dashed lines indicate mean basal synaptic responses. Data are represented as mean ± SEM. *P < 0.05; **P < 0.01. Two-way ANOVA followed by Tukey’s post hoc test (**B**); 1-way ANOVA followed by Tukey’s post hoc test (**D**); 2-tailed unpaired *t* test (**G** and **J**).

Synaptic structural remodeling and functional plasticity are typically highly coordinated (30–33). For example, stress increases spine density and results in the hyperexcitability of neurons in the amygdala and NAc (34). In the chronic pain condition, glutamate neurotransmitter–mediated ACC hyperexcitability coincides with depressive-like behaviors (9). To determine whether chronic pain alters glutamate receptors in ACC neurons, we isolated the postsynaptic density–enriched (PSD-enriched) membrane fraction of ACC neurons 7 weeks after SNI or sham surgery and measured synaptic NMDAR and α-amino-3-hydroxy-5-methyl-4-isoxazolepropionic acid receptor (AMPAR) subunit protein levels with Western blot analysis (35, 36). The synaptic levels of NMDAR subunits GluN1, GluN2A, and GluN2B were increased in SNI-treated mice compared with sham-treated control mice, whereas no differences were detected in AMPAR subunits GluA1 and GluA2 (Figure 5, A and B), suggesting a specific increase in synaptic NMDAR levels. To assess synaptic NMDAR activity, we first measured NMDAR-mediated currents from pyramidal neurons in layer II/III of acutely isolated ACC cortical slices from control mice 7 weeks after sham or SNI surgery. We recorded NMDAR currents elicited by puff application of 100 μm NMDA directly onto ACC pyramidal neurons. We found that chronic neuropathic pain (7 weeks after SNI) markedly increased the amplitude of puff NMDA currents of ACC neurons (Figure 5, C and D), suggesting that NMDAR-mediated activity is enhanced in the ACC in chronic neuropathic pain. LTP is a well-studied cellular model of synaptic plasticity involving synaptic strengthening that is associated with memory formation and chronic pain and requires the activation of NMDARs (37, 38). To determine whether synaptic transmission is increased in ACC neurons in response to chronic neuropathic pain, we performed whole-cell patch-clamp recordings on layer II/III pyramidal neurons from acute ACC slices using a pairing training protocol (80 pulses of presynaptic stimulation at 2 Hz in layer V with postsynaptic depolarization at +30 mV) to trigger NMDAR-dependent LTP in sham-treated and SNI mice (39, 40). Recorded neurons were identified as pyramidal neurons based on morphological features and their ability to show spike frequency adaptation in response to prolonged depolarizing-current injection (41). We found that, although LTP was induced in ACC slices from both sham-treated and SNI mice (7 weeks after surgery), LTP was significantly enhanced in ACC slices from mice subjected to SNI surgery (Figure 5, E–G), indicating that chronic pain significantly increased NMDAR-dependent synaptic potentiation in the ACC. Together, these data suggest that chronic pain–induced depressive symptoms are caused by underlying synaptic structural and functional alterations in ACC neurons.

Since Tiam1 in ACC neurons is required for chronic pain–induced depressive-like behaviors (Figure 2, Figure 3, and Supplemental Figure 6) and Tiam1 is known to couple NMDARs to Rac1-dependent actin dynamics necessary for hippocampal spine and synapse development (13–15), we next investigated whether chronic pain–induced depressive-like behaviors are determined by Tiam1-mediated dendritic spine remodeling and functional synaptic plasticity of ACC neurons. While chronic pain promotes increases in the density of dendritic spines and the F- to G-actin ratio of ACC neurons in control mice, no detectable changes in spine density or F- to G-actin ratio were observed in *Tiam1*-cKO mice (Figure 4, A–C), suggesting that Tiam1 controls chronic pain–induced synaptic structural remodeling of ACC neurons. Similarly, the chronic neuropathic pain–induced increases in synaptic NMDAR subunit protein levels (Figure 5, A and B), synaptic NMDAR currents (Figure 5, C and D), and NMDAR-dependent synaptic potentiation in the ACC (Figure 5, H–J) were attenuated by the conditional deletion of Tiam1 from CaMKIIα-expressing postnatal forebrain excitatory neurons, suggesting that Tiam1 regulates chronic pain–induced synaptic functional plasticity. Together, these results argue that Tiam1 coordinates synaptic structural and functional plasticity of ACC neurons, which underlies ACC hyperactivity and the development of chronic pain–induced depressive-like behaviors.

### Inhibiting Tiam1 signaling alleviates chronic pain–induced depressive-like behaviors.

**Figure 6:**
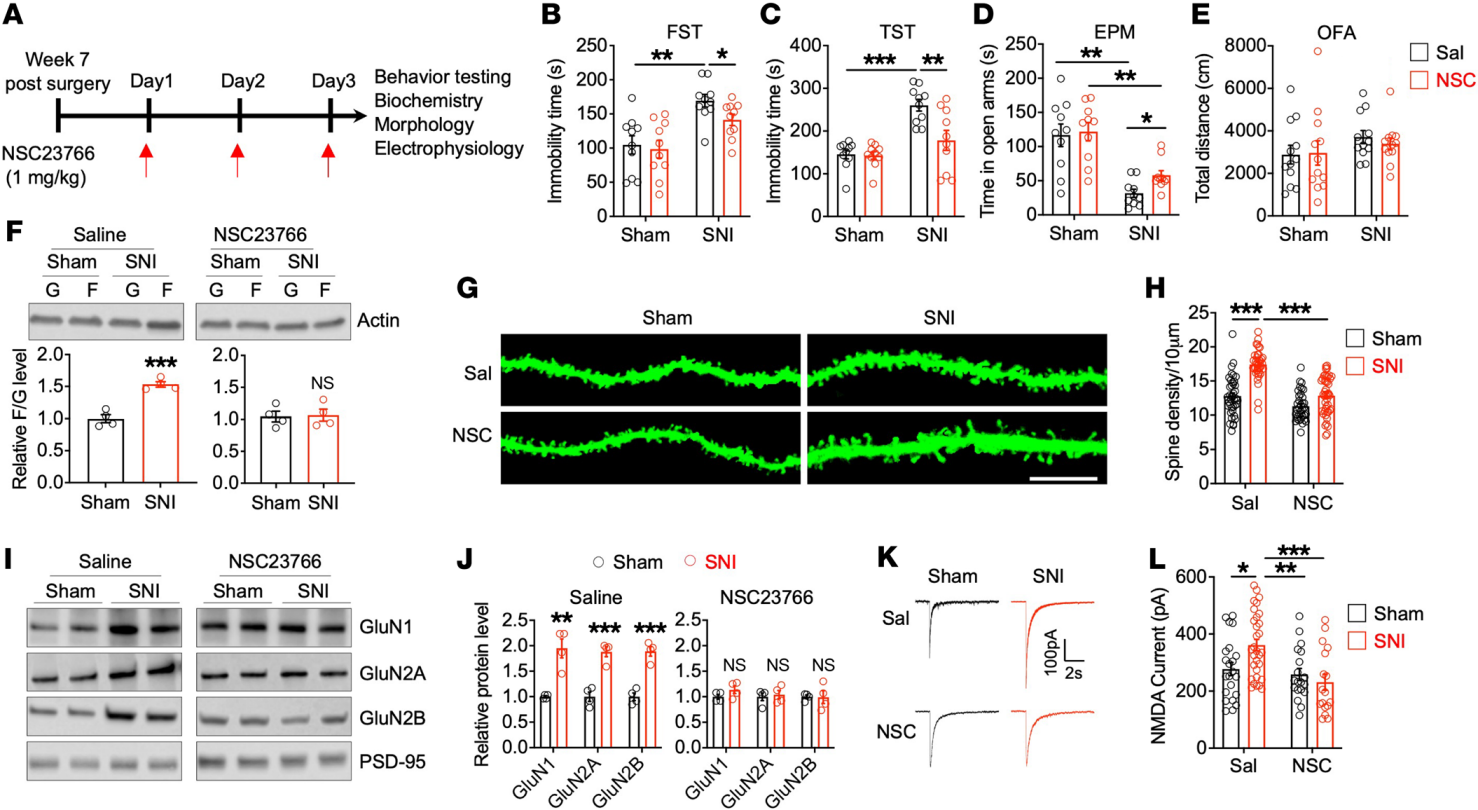
Pharmacological inhibition of Tiam1-Rac1 signaling with NSC23766 alleviates chronic pain–induced depressive-like behaviors and synaptic remodeling. (**A**) Experimental paradigm. (**B**–**E**) Three-day treatment with NSC23766 at 7 weeks after sham or SNI surgery alleviated chronic pain–induced depressive-like behaviors in the FST (**B**) and TST (**C**) and alleviated chronic pain–induced anxiety-like behaviors in the EPM test (**D**), but had no effect on locomotor activity in the OFA test (**E**) (*n* = 10 mice for each group). (**F**–**L**) Three-day treatment with NSC23766 also normalized chronic pain–induced increases in the ratio of F-actin to G-actin (**F**) (*n* = 4 mice for each group), the density of dendritic spines (**G** and **H**) (sham-saline, *n* = 28 dendrites from 3 mice; SNI-saline, *n* = 27 dendrites from 3 mice; sham-NSC23766, *n* = 25 dendrites from 3 mice; sham-NSC23766, *n* = 30 dendrites from 3 mice), the synaptic NMDAR subunit protein levels (**I** and **J**) (*n* = 4 mice for each group), and the NMDAR currents elicited by puff application of 100 μM NMDA to ACC pyramid neurons (**K** and **L**) (sham-saline, *n* = 22 neurons from 4 mice; sham-NSC23766, *n* = 19 neurons from 3 mice; SNI-saline, *n* = 31 neurons from 4 mice; SNI-NSC23766, *n* = 17 neurons from 3 mice). Scale bar: 10 μm. Data are represented as mean ± SEM. *P < 0.05; **P < 0.01; ***P < 0.001. Two-way ANOVA followed by Tukey’s post hoc test (**B**–**E**, **H**, and **I**); 2-tailed unpaired *t* test (sham versus SNI) (**F** and **J**).

Our genetic results indicate that Tiam1 deletion from forebrain excitatory neurons prevents the development of chronic pain–induced depressive-like behaviors and associated synaptic changes in the ACC. Next, we determined whether pharmacological inhibition of Tiam1 signaling could alleviate the depressive-like behaviors and synaptic remodeling induced by chronic pain. NSC23766 is a widely used small molecule Rac1 inhibitor that prevents Rac1 activation by the Rac1-specific GEFs Tiam1 and Trio (42). Three-day treatment with NSC23766 (1 mg/kg, i.p.) applied 7 weeks after SNI did not affect mechanical sensitivity threshold (Supplemental Figure 8), but significantly alleviated SNI-induced depressive-like behaviors, as shown by marked decreased immobility in the FST and TST (Figure 6, A–C). Moreover, NSC23766 treatment reduced SNI-induced anxiety-like behavior in the EPM test (Figure 6D) but did not affect locomotor activity in the OFA test (Figure 6E). Three-day treatment with NSC23766 at 7 weeks after SNI also normalized the F- to G-actin ratio (Figure 6F), the density of dendritic spines (Figure 6, G and H), synaptic NMDAR subunit levels (Figure 6, I and J), and the amplitude of puff NMDAR currents of ACC neurons to sham-treatment levels (Figure 6, K and L). These data suggest that pharmacological inhibition of Tiam1-Rac1 signaling alleviates chronic pain–induced depressive-like phenotypes by normalizing chronic pain–induced synaptic structural and functional remodeling of ACC neurons. A previous study revealed that NSC23766 also acts as a competitive antagonist of muscarinic acetylcholine receptors (mAChRs) within the same concentration range, as it inhibits Rac1 activation by the Rac1 GEFs Tiam1 and Trio (43). Given that mAChR antagonism is known to have antidepressant-like effects in rodents and humans (44, 45), it is possible that NSC23766’s ability to abrogate chronic pain–induced depressive-like behaviors is partly due to mAChR inhibition, but this remains to be determined.

### Ketamine’s sustained antidepressant-like effects in chronic pain are mediated by blocking Tiam1-dependent maladaptive synaptic plasticity in ACC neurons.

**Figure 7:**
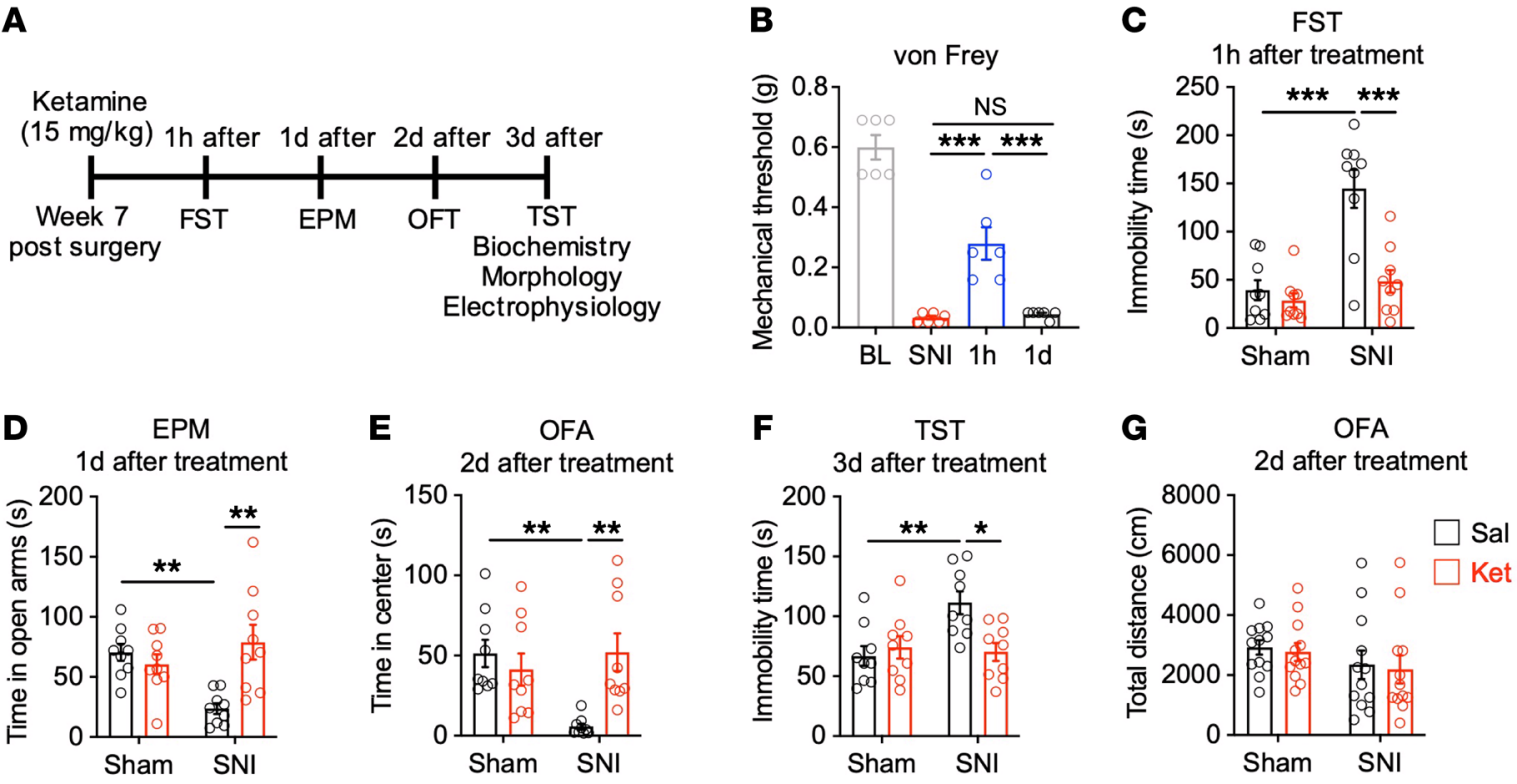
Ketamine reduces depressive- and anxiety-like behaviors in mouse models of chronic pain. (**A**) Experimental paradigm. (**B**) A single ketamine dose relieved SNI-induced mechanical hypersensitivity at 1 hour, but not 1 day after administration (*n* = 6 mice). (**C**) FST showing a decrease in the immobility time of SNI mice 1 hour after ketamine injection (*n* = 9 mice for each group). (**D**) Ketamine resulted in an increased time in the open arms of SNI animals in the EPM test 1 day after treatment (*n* = 9 mice for each group). (**E**) Ketamine-treated SNI mice showed an increased time in center during the OFA 2 days after administration (*n* = 9 mice for each group). (**F**) Ketamine reduced immobility times of SNI mice in the TST 3 days after administration (*n* = 9 mice for each group). (**G**) Ketamine had no effect on locomotor activity in the OFA 2 days after administration (*n* = 12 mice for each group). Data are represented as mean ± SEM. *P < 0.05; **P < 0.01; ***P < 0.001. One-way ANOVA followed by Tukey’s post hoc test (**B**); 2-way ANOVA followed by Tukey’s post hoc test (**C**–**G**).

The NMDAR antagonist ketamine has both analgesic and antidepressant properties (46, 47). A single subanesthetic dose of ketamine produces rapid and sustained antidepressant-like effects in chronic pain–induced depression without decreasing sensory hypersensitivity (10, 11). While these features have revived interest in ketamine as a promising treatment for comorbid depression in chronic pain, the mechanism by which ketamine mediates its effects is not fully understood. To further characterize ketamine’s effects on chronic pain–induced depressive-like behaviors, we performed sham and SNI surgery on WT mice. Seven weeks following surgery, we examined the time response of a single dose of ketamine (15 mg/kg, i.p.) on mechanical sensitivity. We found that ketamine alleviated the decreased mechanical threshold observed in SNI animals 1 hour after administration, but this effect was no longer present at 24 hours after treatment (Figure 7, A and B), suggesting that the antiallodynic effect of ketamine is transient. In contrast with its antiallodynic effects, we found that a single injection of a subanesthetic dose of ketamine (15 mg/kg, i.p.) was sufficient to reduce neuropathic pain–induced depressive-like behaviors for at least 3 days (Figure 7, A and C–F). Specifically, animals with neuropathic pain showed a decrease in depressive/anxiety-like behaviors 1 hour after ketamine administration in the FST (Figure 7C), 1 day after ketamine administration in the EPM test (Figure 7D), 2 days after ketamine administration in the OFA test (Figure 7E), and 3 days after ketamine administration in the TST (Figure 7F). No difference in locomotor activity was observed between saline and ketamine groups in the OFA test 2 days after drug treatment (Figure 7G). These data are consistent with previous reports that ketamine induces rapid and sustained antidepressant-like effects in chronic pain–induced depressive-like behaviors (10, 11).

**Figure 8:**
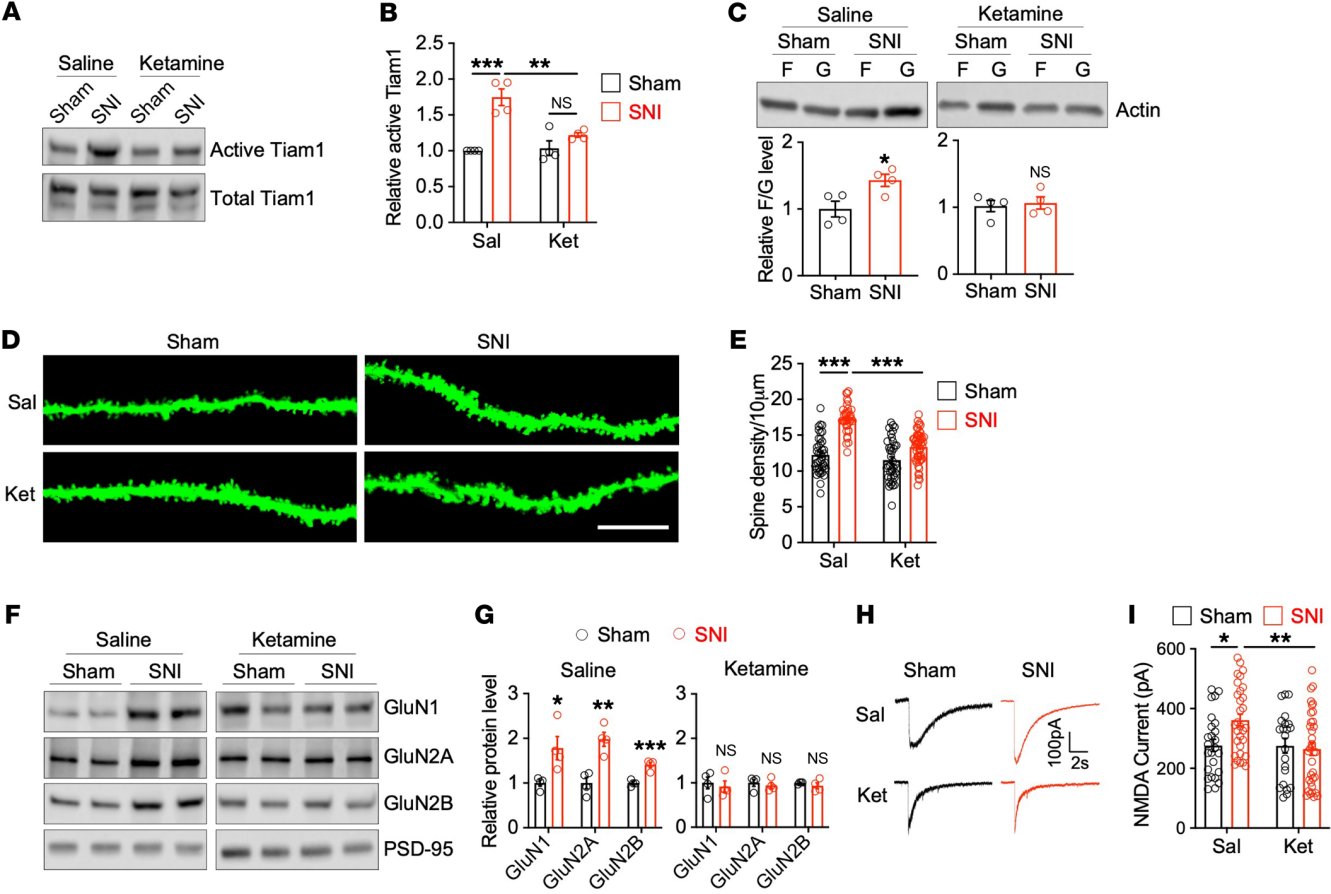
Ketamine blocks Tiam1-mediated synaptic plasticity in ACC neurons of mouse models of chronic pain. (**A** and **B**) Ketamine treatment blocked chronic pain–induced increases in Tiam1 activity in the ACC, as demonstrated by the GST-Rac1G15A affinity-precipitation assay (*n* = 4 mice for each group). (**C**) A single subanesthetic dose of ketamine (15 mg/kg) blocked chronic pain–induced increases in the ratio of F-actin to G- actin in the ACC (*n* = 4 mice for each group). (**D** and **E**) Ketamine treatment prevented chronic pain–induced increases in the density of dendritic spines in ACC neurons (sham-saline, *n* = 25 dendrites from 3 mice; sham-ketamine, *n* = 25 dendrites from 3 mice; SNI-saline, *n* = 26 dendrites from 3 mice; SNI-ketamine, *n* = 30 dendrites from 3 mice). Scale bar: 10 μm. (**F** and **G**) Ketamine treatment abolished chronic pain–induced increases in synaptic NMDAR subunit protein levels in the ACC (*n* = 4 mice for each group). (**H** and **I**) Ketamine treatment blocked chronic pain–induced increases in the NMDAR currents elicited by puff application of 100 μM NMDA to ACC pyramid neurons (sham-saline, *n* = 25 neurons from 4 mice; sham-ketamine, *n* = 22 neurons from 3 mice; SNI-saline, *n* = 31 neurons from 4 mice; SNI-ketamine, *n* = 35 neurons from 4 mice). Data are represented as mean ± SEM. *P < 0.05; **P < 0.01; ***P < 0.001. Two-way ANOVA followed by Tukey’s post hoc test (**B**, **E**, and **I**); 2-tailed unpaired *t* test (sham versus SNI) (**C** and **G**).

Since Tiam1 is activated in the ACC of mouse models of chronic pain, where it is required for chronic pain–induced synaptic plasticity and depressive-like behaviors (Figures 1, 2, 3, and 4), we next asked whether ketamine’s sustained antidepressant-like effects in chronic pain may be mediated, at least in part, by blocking Tiam1 function. To address this question, we first examined the effect of ketamine treatment on Tiam1 activity in the ACC of mouse models of chronic pain. Three days after a single dose of 15 mg/kg (i.p.) ketamine or saline treatment on sham or mouse models of chronic pain (7 weeks after SNI surgery), we performed an active GEF affinity-precipitation assay using GST-Rac1G15A on ACC homogenates. We found that ketamine treatment attenuated chronic pain–induced Tiam1 activity in the ACC (Figure 8, A and B), indicating that a single ketamine administration blocks Tiam1 activation in the ACC of SNI mice. Moreover, we found that 3 days after a single-dose ketamine treatment (15 mg/kg, i.p.), chronic pain–induced increases in the F- to G-actin ratio, density of dendritic spines, synaptic NMDAR subunit levels, and amplitude of puff NMDAR currents in ACC neurons were normalized to levels in sham-treated uninjured mice (Figure 8, C–I). These data suggest that ketamine’s sustained antidepressant-like effects in chronic pain–induced depressive-like behaviors may be mediated in part by blocking Tiam1-dependent synaptic structural and functional plasticity in ACC neurons.

## Discussion

**Figure 9:**
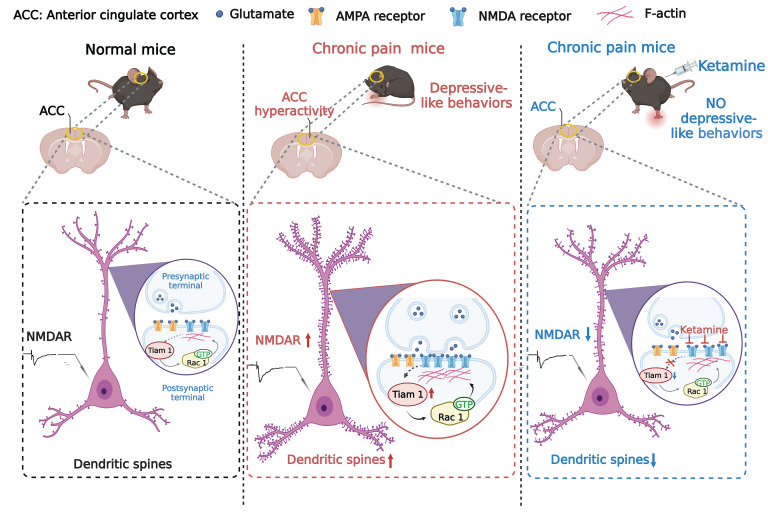
Proposed model. Tiam1 links chronic pain–stimulated NMDARs to Rac1 activation in the ACC that orchestrates synaptic structural plasticity via actin and spine remodeling and functional plasticity via synaptic NMDAR stabilization, which contributes to ACC hyperactivity and depressive-like behaviors. Ketamine relieves depressive-like behaviors resulting from chronic pain by blocking Tiam1-mediated maladaptive plasticity in the ACC.

We have provided multiple lines of evidence to support the idea that Tiam1 mediates chronic pain–induced synaptic structural and functional plasticity in ACC neurons via actin cytoskeleton remodeling and synaptic NMDAR stabilization, which promotes ACC hyperactivity and depressive-like behaviors. Moreover, our results suggest that the sustained antidepressant-like effects of ketamine in chronic pain–induced depressive-like behaviors are mediated, at least in part, by targeting Tiam1-dependent synaptic plasticity in ACC neurons (Figure 9). We previously identified Tiam1 as a critical mediator of NMDAR-dependent dendritic spine development (13). Here, in addition to showing that Tiam1 is required for chronic pain–induced spinogenesis in adult mice, we demonstrated that Tiam1 enhances synaptic NMDAR expression in ACC neurons, promoting ACC hyperactivity that drives the emotional consequences of chronic pain (9). Depressive-like behaviors induced by chronic pain often persist for weeks after recovery from mechanical hypersensitivity (9). Tiam1 likely contributes to the long-term nature of chronic pain–induced depressive-like behaviors by mediating synaptic structural and functional plasticity of ACC neurons, which results in persistent modifications of ACC neuron synaptic connectivity (48, 49).

NMDARs play a pivotal role in ACC hyperactivity that drives comorbid depressive-like behaviors in chronic pain (9) as well as in spinal dorsal horn hyperactivity responsible for hyperalgesia and allodynia (50, 51). As an NMDAR antagonist, ketamine’s rapid antinociceptive and antidepressant-like actions in chronic pain–induced depression (Figure 7) might be achieved by inhibiting NMDAR-mediated sensitization of spinal dorsal horn and ACC neurons. The Rho GTPase Rac1 promotes the formation, growth, and stabilization of spines and synapses by controlling actin cytoskeleton organization (12). We previously identified Tiam1 as a critical regulator of Rac1-dependent spine morphogenesis in brain development and showed that Tiam1 is activated by synaptic NMDARs and mediates their effects on actin and spine remodeling (13). Our data indicate that during the development of comorbid depression, Tiam1 is activated in response to chronic pain–stimulated NMDARs in the ACC and links NMDARs to Rac1 activation that orchestrates synaptic structural plasticity via actin and spine remodeling and functional plasticity via synaptic NMDAR stabilization, which contributes to ACC hyperactivity and depressive-like behaviors. Ketamine treatment likely reduces Tiam1 activation by inhibiting NMDAR activation and thereby blocking Rac1-dependent actin polymerization. Thus, ketamine may mediate its sustained antidepressant-like effects in part by inhibiting Tiam1-mediated maladaptive synaptic structural and functional plasticity in ACC neurons, which likely underlies chronic pain–induced depressive-like behaviors (Figure 9).

Clinical and preclinical studies have shown that, despite similar behavioral symptoms, the mechanisms underlying chronic pain–induced depression and stress-induced depression are distinct at synaptic and circuit levels (52–55). Whereas neuronal atrophy and synaptic loss in the PFC and hippocampus are hypothesized to cause stress-induced depression (56), our data and others suggest that spinogenesis and synaptic potentiation in the ACC promote chronic pain–induced depressive-like behavior (8, 9). Rac1 signaling has also been implicated in stress-induced depression (57, 58) and the antidepressant-like effects of ketamine (58). However, contrary to our findings that ketamine treatment reduces chronic pain–induced depressive-like behaviors by blocking increases in Tiam1-Rac1 activation, dendritic spine density, and synaptic NMDAR levels and function in the ACC, ketamine was shown to improve depression-like behaviors in stressed rats by upregulating Rac1 activity and increasing dendritic spine density and synaptic-related protein expression in the hippocampus (58). In both cases, stress and chronic pain appear to drive alterations in Tiam1-Rac1 signaling and synapse connectivity and function that are rescued by ketamine treatment, but the manner of change and the brain regions/neural circuits involved may differ. More research is required to elucidate the distinct mechanisms of stress- and chronic pain–induced depression and the effects of ketamine on these conditions. For instance, brain-derived neurotrophic factor (BDNF) and mTOR signaling have been implicated in the antidepressant-like effects of ketamine in models of stress-induced depression (59, 60), but whether they also contribute to ketamine’s antidepressant-like effects in chronic pain–induced depression and how they interact with Tiam1-mediated signaling and synaptic plasticity remain to be determined. It is also interesting to note that a recent study found that early life inflammation promotes stress-induced depressive symptoms in adolescence via microglial engulfment of dendritic spines in the ACC (4). Given our results showing that ACC Tiam1 mediates inflammatory pain–induced depressive-like behavior, it is possible that Tiam1-mediated alteration in wiring/function of ACC glutamatergic neurons may be involved in the development of depressive symptoms caused by early life inflammation, but more research needs to be done.

Besides the ACC, additional brain regions, such as the amygdala, NAc, insular cortex, and PFC, are thought to contribute to the comorbidity between pain and mood disorders (21). Interestingly, while deleting Tiam1 from ACC neurons prevented chronic pain–induced depressive-like behaviors, it had no effect on chronic pain–induced anxiety-like behaviors (Figure 3, E–H). In contrast, deletion of Tiam1 from postnatal forebrain excitatory neurons prevented both depressive- and anxiety-like behaviors in mouse models of chronic pain (Figure 2, D–H). Accumulating evidence suggests that, besides the ACC, the amygdala is another key neural substrate for the interactions between pain and negative affective states (61–64). In particular, the central amygdala (CeA) is a strong candidate brain region for mediating anxiety-like behaviors resulting from chronic pain, given the amygdala’s well-documented role in anxiety (65), the involvement of activated CeA neurons in itch-related anxiety-like behavior (66), and the function of NMDAR-mediated hyperactivity and increased CeA plasticity in pain and emotional processing (67, 68). Further research is needed to determine whether Tiam1 mediates synaptic plasticity in the CeA driving chronic pain–induced anxiety. Our work demonstrates the critical role Tiam1 plays in the pathophysiology of chronic pain–induced mood dysregulation and the sustained antidepressant-like effects of ketamine, revealing it as a potential therapeutic target for the treatment of comorbid mood disorders in chronic pain.

## Methods

### Animals

For cKO of *Tiam1* from postnatal forebrain excitatory neurons, *Tiam1^fl/fl^* mice, generated as described (15), were crossed with CaMKIIα-Cre mice, and the resulting CaMKIIα-Cre:Tiam1^+/fl^ mice were then crossed with *Tiam1^fl/fl^* mice to obtain CaMKIIα-Cre:*Tiam1^fl/fl^* (*Tiam1*-cKO mice) as well as *Tiam1^fl/fl^* littermates (control) for use in experiments. Genotyping of *Tiam1* mice was determined by PCR from tail DNA using the following primers: P1, ACGTGTGTTAATTAGCCAGGTTTGATGG; P2, GATCCACTAGTTCTAGAGCGGCCGAA; P3, CTACCCGGAGGAAGTGGAAGCACTACT. Genotyping of CaMKIIα-Cre mice was determined by PCR from tail DNA using the following primers: forward, GCATTACCGGTCGATGCAACGAGTGATGAG; reverse, GAGTGAACGAACCTGGTCGAAATCAGTGCG. *Tiam1^fl/fl^* mice were maintained on a 129SvEv background, while CaMKIIα-Cre mice were maintained on a C57BL/6J background. All experiments used age-matched male and female mice.

### Pain models

#### Neuropathic pain.

Neuropathic pain was induced by the SNI model (18). Briefly, mice were anesthetized with 2% isoflurane. A heating pad was used to maintain the core body temperature of the animals at 37°C. An incision was made on the left lateral thigh to expose the sciatic nerve. We ligated and sectioned the common peroneal and tibial nerves (leaving the sural nerve intact) with a 5-0 silk suture under a surgical microscope. The sham procedure consisted of the same surgery without nerve ligation and section.

#### Inflammatory pain.

CFA (10 μl, Sigma-Aldrich) was injected into the plantar surface of the left hind paws of mice using an insulin syringe (29 gauge) under brief isoflurane anesthesia to induce persistent inflammatory pain. The persistence of inflammatory pain was ensured by a second CFA (10 μl) injection on the tenth day. Saline (0.9% NaCl) was injected as control.

### Viral injection

Mice were anesthetized with 2% isoflurane and placed in a stereotaxic frame (Kopf). A heating pad was used to maintain the core body temperature of the animals at 37°C. The coordinates were defined as dorsal-ventral (DV) from the brain surface, anterior-posterior (AP) from bregma, and medio-lateral (ML) from the midline. A volume of 1 μl rAAV8-hSyn-GFP or rAAV8-hSyn-Cre-GFP (UNC Vector Core) was injected bilaterally into the ACC (areas 24a/24b, AP, 0.7 mm; ML, ±0.3 mm; DV, −1.5 mm) using a glass micropipette attached to a Hamilton microsyringe connected to an infusion pump at a rate of 200 nl/min (9). After injection, the microelectrodes remained in place for 10 minutes and then the skin was sutured.

### Behavioral assessments of nociception

For all behavioral tests, the experimenters performing the behavioral tests and quantitative analyses were blinded to mouse genotypes and treatments.

#### Classification of mouse behaviors into reflexive and affective-motivational nociceptive responses.

The reflexive and affective-motivational nociceptive responses in mice were classified based on previous reports (69–71). Briefly, a cutaneous noxious stimulus can elicit several distinct behavioral responses: (a) withdrawal reflexes, rapid reflexive withdraws of the paw that occur in response to noxious stimulus but cease once the stimulus is removed; (b) affective-motivational responses, temporally delayed (relative to the noxious stimulation), directed licking and biting of the paw (termed “attending”), extended lifting or guarding of the paw, and/or escape responses characterized by hyperlocomotion, jumping away from the noxious stimulus, or rearing. Paw withdrawal reflexes are observed in decerebrate rodents only while the stimulus is in contact with tissue, but immediately cease once the stimulus is removed. These reflexes are classically measured in studies of hypersensitivity and involve the spinal cord and brain stem circuits (72). In contrast, affective-motivational responses are complex behaviors that indicate the subject’s motivation and arousal to make the aversive sensations cease by licking the affected tissue, protecting the tissue, or seeking an escape route. The affective-motivational responses require processing by limbic and cortical circuits in the brain (73–76).

#### Mechanical reflexive assays.

To evaluate mechanical reflexive sensitivity, we applied a series of calibrated von Frey filaments (Stoelting). These filaments were applied perpendicularly to the plantar surface of the hind paw with sufficient force to bend the filament. Rapid withdrawal of the paw away from the stimulus within 4 seconds was characterized as a positive response. If there was no response, the filament of the next greater force was applied. After a response, the filament of the next lower force was applied. We calculated the tactile stimulus force that produced a 50% likelihood of a withdrawal response using the up-down method (36, 77).

#### Thermal affective assays.

To evaluate affective-motivational responses evoked by thermal stimulation, we applied a single, unilateral 50 μl drop of acetone (evaporative cooling) to the left hind paw, and the duration of attending behavior was collected for up to 60 seconds after the stimulation. To prevent behavioral sensitization that can result from multiple noxious stimulations, the animals were only treated once with acetone for a given testing session (70, 71).

#### MCA assay.

Voluntary aversion to a noxious stimulus was assessed using a commercial 3-chambered apparatus, the Mechanical Conflict-Avoidance System (Noldus). The Mechanical Conflict-Avoidance System contains an aversive lighted area, a walkway with mechanical probes, and an attractive dark area. The mice were placed on the aversive lighted area and given the opportunity to escape from this area to the preferred dark area through the walkway. The walkway consists of mechanical probes that are painful, but not sharp enough to cause any tissue damage when walked on. Longer latencies to escape the light chamber indicate increased motivation to avoid the probes, and this escape latency is the measure of pain-related behavior in this test. We performed the operant MCA test on mice with modifications, as recently described in detail (23, 24). The test was performed for 2 days. Before the testing day, mice were acclimated to the MCA unit for 5 minutes with the LEDs switched off, the barrier door open, and the mechanical probe height set to zero. For testing, mice were placed into the lighted chamber with the lid closed. The LEDs were switched on after 10 seconds, and the barrier was removed 20 seconds thereafter. The behavior of each mouse was recorded, starting from its introduction into the lighted chamber until the mouse crossed the halfway point of the walkway with mechanical probes, within a 120-second cutoff period. After running all mice in a particular cohort at a probe height of 0 mm, the process was repeated with the probe height set to 5 mm. The duration between the barrier opening and the mouse crossing the midpoint of the walkway with mechanical probes was quantified and expressed as escape latency.

Behavioral assessment of depressive- and anxiety-like behaviors

For all behavioral tests, the experimenters performing the behavioral tests and quantitative analyses were blinded to mouse genotypes and treatments. Behavioral assessments were performed during the light phase, between 9:00 am and 5:00 pm. Mice were habituated at least 1 day in the testing room before testing. On the test day, mice were transferred to the test room and were left undisturbed for at least 30 minutes prior to the start of testing. White noise (~60 dB) was present throughout the adaptation to the room and test.

#### Rotarod.

To examine baseline motor behavior, mice were subjected to an accelerating rotarod test on 2 consecutive days with 4 trials per day. Mice rested at least 30 minutes between trials. The rotation speed of the rotarod increased from 4 to 40 rpm during the test. The duration of time the mice stayed on the rotarod (latency to fall) was recorded in seconds, and all 8 trials were analyzed.

#### OFA.

Mice were placed in the center of an open-field arena (40 × 40 cm) and movement was recorded for 30 minutes with a Versamax computer-assisted tracking system (Accuscan Inc.). The total distance traveled was used as a measure of locomotion. The ratio between the distance traveled in a 20 × 20 cm square in the center and the total distance traveled was calculated and used as a measure of anxiety-like behavior. The area was cleaned with 75% ethanol after each test to remove olfactory cues from the apparatus.

#### EPM.

Anxiety-like behavior was measured in the EPM test for 10 minutes. Briefly, mice were placed into a maze with two 25 × 7 cm corridors with 15 cm high walls and two corridors with no walls, connected by a central square. The maze stood 50 cm above the floor. Time spent and percentages of entries into the open arms, which are measures of anxiety-like behavior, were recorded using the ANY-MAZE system (Stoelting). The area was cleaned with 75% ethanol after each test to remove olfactory cues from the apparatus.

#### SPT.

Mice were habituated to drinking from 2 bottles for 3 days before testing and housed individually 4 hours before the dark cycle with free access to food. The SPT was conducted during the 12 hours of the dark cycle, and during the test, mice were presented with 2 bottles, one containing water and the other containing 1% sucrose. Water and sucrose solution intake were measured, and the preference for sucrose was calculated by dividing the weight of 1% sucrose intake by the total weight of water and 1% sucrose intake.

#### TST.

The TST was performed to study depressive-like behavior. Mice were taped by the tail to a metal bar connected to a transducer that transmitted movements to a computer. The time of immobility during a 6-minute test was calculated using the ANY-maze System. The area was cleaned with 75% ethanol after each test to remove olfactory cues from the apparatus.

#### FST.

The FST was performed as described with minor modifications. In brief, individual mice were forced to swim for 6 minutes in a transparent plastic vessel (diameter 26 cm, height 50 cm) filled with 30 cm of water (22 ± 1°C). Immobility time was counted during a test period of 6 minutes using the ANY-maze System. Immobility time was defined as the duration a mouse was floating in the water without struggling and was making only small movements to keep its head above the water.

### Biochemical assays

#### Affinity-precipitation assay for Tiam1 activity.

Tiam1 activity was measured using an affinity precipitation assay previously described (78). Briefly, The ACC from treated mice was isolated, homogenized in cold lysis buffer (25 mM HEPES, pH 7.4, 0.1 M NaCl, 1% NP40, 5 mM MgCl_2_, 10% glycerol, 1 mM DTT, 10 μg/ml leupeptin, 10 μg/ml aprotinin, and 1 mM sodium orthovanadate), and centrifuged at 15,000*g* for 30 minutes. The supernatant was incubated with 30 μg of GST-Rac1G15A bound to GSH-agarose beads for 2 hours at 4°C and mixed gently on a rocking shaker. After washing with lysis buffer 3 times, beads were resuspended in Laemmli buffer. Samples were resolved by SDS-PAGE, transferred to nitrocellulose membrane, which was blocked with 5% fat-free milk in 0.1% Tween-PBS, and incubated with anti-Tiam1 (1:1000). Active Tiam1 was determined by Western blot analysis from the precipitated fraction and normalized to total protein (input).

#### F-actin to G-action ratio.

The F-actin to G-actin ratio was determined by Western blot, as previously described (79, 80). Briefly, the two forms of actin differ in that F-actin is insoluble, whereas G-actin is soluble. The ACC from sham- or SNI-treated control and *Tiam1*-cKO mice was isolated, homogenized in cold lysis buffer (10 mM K_2_HPO_4_, 100 mM NaF, 50 mM KCl, 2 mM MgCl_2_, 1 mM EGTA, 0.2 mM DTT, 0.5% Triton X-100, 1 mM sucrose, pH 7.0), and centrifuged at 15,000*g* for 30 minutes. Soluble actin (G-actin) was measured in the supernatant. The insoluble F-actin in the pellet was resuspended in lysis buffer plus an equal volume of buffer 2 (1.5 mM guanidine hydrochloride, 1 mM sodium acetate, 1 mM CaCl_2_, 1 mM ATP, 20 mM Tris-HCl, pH 7.5) and incubated on ice for 1 hour to convert F-actin into soluble G-actin, with gentle mixing every 15 minutes. The samples were centrifuged at 15,000*g* for 30 minutes, and F-actin was measured in this supernatant. Samples from the supernatant (G-actin) and pellet (F-actin) fractions were proportionally loaded and analyzed by Western blotting. The ratio of F- to G-actin from the sham-treated group was used as standard 1, and the fold change ratio from the SNI group was calculated.

#### Synaptosome preparation.

Synaptosome preparation was performed as in our previous publications (36). The ACC from sham- or SNI-treated mice was homogenized using glass-Teflon homogenizer in 10 volumes of ice-cold HEPES-buffered sucrose (0.32 M sucrose, 1 mM EGTA, and 4 mM HEPES at pH 7.4) containing a protease inhibitor cocktail (Sigma-Aldrich). The homogenate was centrifuged at 1,000*g* for 10 minutes at 4°C to remove the nuclei and large debris. The supernatant was centrifuged at 10,000*g* for 15 minutes to obtain the crude synaptosome fraction. The synaptosome pellet was lysed via hypoosmotic shock in 9 volumes of ice-cold HEPES buffer with the protease inhibitor cocktail for 30 minutes. The lysate was centrifuged at 25,000*g* for 20 minutes at 4°C to obtain the synaptosome membrane fraction for the following immunoblotting experiments.

#### Single-cell dissociation and flow cytometry.

Dissociation of the ACC neurons was performed with the Adult Brain Dissociation Kit (Miltenyi Biotec) with modification. Briefly, ACC tissue chunks were incubated in 1 ml of enzyme P solution for 1 hour at 37°C and 5% CO_2_. After 10 minutes of incubation, tissues were triturated briefly with a P1000 pipette tip and returned. Cells were triturated another 4 times (around 30 times each) with a P200 pipette tip over the rest of the remaining incubation time. At room temperature, cell suspensions were centrifuged at 350*g* for 10 minutes, resuspended in 1 ml PBS with proteinase inhibitor cocktail (Sigma-Aldrich), and centrifuged again. Supernatant was removed and 1 ml PBS with proteinase inhibitor cocktail was added to cells. Cells were passed through a 70 μm cell strainer to remove debris. Samples were centrifuged (350*g* for 8 minutes at 4°C) and resuspended in 0.5 ml of PBS with proteinase inhibitor cocktail and kept on ice for flow cytometry. Cells were sorted via the Sony SH800 Cell Sorter. GFP-positive and -negative cells were used for subsequent immunoblotting experiments.

#### Immunoblotting.

The protein samples were homogenized in RIPA buffer containing 50 mM Tris-HCl (pH 7.4), 1% NP-40, 0.1% SDS, 150 mM NaCl, 1 mM EDTA, 1 mM Na_3_VO_4_, and 1 mM NaF in the presence of a proteinase inhibitor cocktail (Sigma-Aldrich). Lysates were centrifuged at 18,000*g* for 30 minutes at 4°C. The supernatant was carefully collected, and protein concentration was measured using a DC Protein Assay Kit (Bio-Rad). A total of 30 μg of the total proteins from each sample was loaded and separated using 4% to 15% Tris-HCl SDS-PAGE gels. The resolved proteins were transferred to an Immobilon-P membrane (Millipore). The membrane was treated with 5% nonfat dry milk in TBST at 25°C for 1 hour and then incubated in TBST supplemented with 0.1% Triton X-100 and 1% BSA and primary antibodies overnight at 4°C. The membrane was washed 3 times and then incubated with horseradish peroxidase–conjugated secondary antibodies for 1 hour at 25°C. The protein band was revealed using an ECL Plus Detection Kit (Thermo Fisher Scientific), and protein band density was quantified with the Odyssey Fc Imager (LI-COR Biosciences) and normalized to the control protein band on the same blot. Tiam1 was detected using rabbit anti-Tiam1 antibody (sc-872, 1:1,000; Santa Cruz Biotechnology Inc.) or sheep anti-Tiam1 antibody (AF5038, 1:1,000; R&D Systems). Actin was detected using mouse anti-actin antibody (MAB1501, 1:10,000; Millipore). GluN1 was detected using rabbit anti-GluN1 antibody (G8913, 1:1,000; MilliporeSigma). GluN2A was detected using rabbit anti-GluN2A antibody (PA5-35377, 1:1,000; Thermo Fisher Scientific). GluN2B was detected using anti-mouse GluN2B antibody (75-002, 1:1,000; NeuroMab). GluA1 was detected using mouse anti-GluA1 antibody (75-327, 1:1,000; NeuroMab). GluA2 was detected using rabbit anti-GluA2 antibody (ab10529, 1:1,000; Millipore). PSD-95 was detected using rabbit anti–PSD-95 antibody (ab18258, 1:2,000; abcam). GAPDH was detected using mouse anti-GAPDH antibody (sc-47724, 1:1,000; Santa Cruz Biotechnology Inc.).

### Morphological analysis

To observe the effects of Tiam1 on spine remodeling in the ACC, rAAV8-hSyn-EGFP (UNC vector core) was injected bilaterally in the ACC and was used to specifically label the neurons. ACC sections (40 μm thick) were collected from mice perfused with 4% PFA, and only dendritic spines on neurons labeled with EGFP were selected for spine analysis in a blinded manner, as previously described (15). All spine images were captured using a Laser Scanning Confocal Microscope (LSCM, Zeiss LSM 880) with a ×63 oil 210 immersion objective. Z series images were taken at an interval of 0.37 μm for each dendrite. Spine morphometric analysis was done in a blinded manner using Imaris software (Bitplane Scientific Software) as previously described (81).

### Brain slice preparation and electrophysiology

The brain slices containing the ACC were obtained following a previous protocol (82) with some modification. In brief, mice were anesthetized with 3% isoflurane and decapitated. Their brains were rapidly removed and collected into ice-cold (~0°C) oxygenated *N*-methyl-d-glutamine (NMDG) solution containing 93 mM NMDG, 93 mM HCl, 2.5 mM KCl, 1.2 mM NaH_2_PO_4_, 30 mM NaHCO_3_, 20 mM HEPES, 25 mM glucose, 5 mM sodium ascorbate, 2 mM thiourea, 3 mM sodium pyruvate, 10 mM MgSO_4_, and 0.5 mM CaCl_2_, pH 7.35 (all from Sigma-Aldrich). Coronal slices were cut 300 μm thick using a Leica VT1200 microtome following coordinates provided in the Allen Brain Atlas for adult mice (http://atlas.brain-map.org). The slices were subsequently incubated at 34.0 ± 0.5°C in oxygenated NMDG solution for 10 to 15 minutes before being transferred to the artificial cerebrospinal fluid (ACSF) solution containing 125 mM NaCl, 2.5 mM KCl, 1.25 mM NaH_2_PO_4_, 25 mM NaHCO_3_, 1 mM MgCl_2_, 11.1 mM glucose, and 2 mM CaCl_2_, pH 7.4 (all from Sigma-Aldrich) for about 30 minutes. The slices were allowed to recover in ACSF equilibrated with bubbling with a 95%O_2_/5%CO_2_ gas mixture at room temperature (approximately 25°C) for at least 1 hour before experiments. During the recordings, individual slices were transferred to a customized recording chamber and submerged in a chamber continuously perfused with oxygenated ACSF warmed to 32–34°C by passing it through a feedback-controlled in-line heater (Temperature Controller VII, Luigs & Neumann GmbH). Recorded cells were generally located in layer II/III.

For the puff NMDA current, the pipette internal solution contained 135.0 mM potassium gluconate, 5.0 mM TEA, 2.0 mM MgCl_2_, 0.5 mM CaCl_2_, 5.0 mM HEPES, 5.0 mM EGTA, 5.0 mM Mg-ATP, 0.5 mM Na-GTP, and 10 mM lidocaine (lignocaine) *N*-ethyl bromide (adjusted to pH 7.2–7.4 with 1M KOH; 290–300 mOsmol/L). NMDAR-mediated currents were elicited by puff application of 100 μM NMDA to the recorded neuron at a holding potential of –60 mV. Positive pressure (4 p.s.i., 15 ms; Picospritzer III) was applied, and puff application of the vehicle produced no currents. The tip of the puff pipette was placed approximately 100 to 150 μm away from the recorded neurons in the presence of 1 μm TTX. To minimize the Mg^2+^ block of NMDARs, the puff NMDA currents were recorded in an extracellular solution containing no Mg^2+^ and 10 μM glycine (36, 83).

NMDAR-mediated excitatory postsynaptic currents (EPSCs) were recorded from layer II/III neurons using a HEKA amplifier, and stimulations were delivered using a stimulating electrode placed in layer V of the ACC (40). EPSCs were induced by repetitive stimulations at 0.05 Hz, and neurons were voltage clamped at −70 mV. The recording pipettes (3–5 MΩ) were filled with solution containing 121 mM K-gluconate, 4 mM KCl, 10 mM HEPES, 4 mM Mg-ATP, 0.3 mM Na_3_-GTP, 10 mM Na_2_-phosphocreatine, and 13.4 mM biocytin (all from Sigma-Aldrich), adjusted to pH 7.2 with KOH. After obtaining stable EPSCs for 10 minutes, LTP was induced by 80 paired presynaptic pulses at 2 Hz with postsynaptic depolarization at +30 mV in layer V (39). Picrotoxin (100 mM) was always present to block GABAA receptor–mediated inhibitory synaptic currents. The access resistance was 1 to 30 MΩ and was monitored throughout the experiment. Data were discarded if access resistance changed by more than 15% during an experiment.

### Statistics

All statistical analyses were performed using Prism software, version 9 (GraphPad Software Inc.). No statistical methods were used to predetermine sample sizes, but our sample sizes were similar to those reported in previous publications (36, 51). Normality was measured by the Shapiro-Wilk test. Data that met these 2 conditions were analyzed using a 2-tailed unpaired or paired *t* test, 1-factor ANOVA, and repeated-measures ANOVA followed by Tukey’s multiple comparisons test. Data are represented as mean ± SEM. All behavioral, electrophysiological, biochemical, and morphological data were obtained by counterbalancing experimental conditions with controls. We did not find any significant differences between male and female animals in this study, and all presented data are the pooled data from both sexes. Statistical significance was defined as *P* < 0.05.

### Study approval

All animal protocols were approved by the Institutional Animal Care and Use Committee of Baylor College of Medicine and were in accordance with NIH guidelines.

## Author contributions

QR performed behavioral testing and morphological characterization analyses and analyzed and interpreted data. YL performed slice recordings and analyzed and interpreted data. ABS performed viral injections. FAB assisted with morphological characterization analyses. CY assisted with animal management and behavioral testing. JPC assisted with ketamine experiment design. DPL assisted with recording experiment design, interpreted data, and revised the article. KFT designed the project, interpreted data, and revised the article. LL conceived and designed the project, performed biochemical experiments and animal surgeries, analyzed and interpreted data, and drafted and edited the article.

## Supplementary Material

Supplemental data

## Version 1 12/15/2022

Electronic publication

## References

[1] Bair MJ (2003). Depression and pain comorbidity: a literature review. Arch Intern Med.

[2] Zhou W (2019). A neural circuit for comorbid depressive symptoms in chronic pain. Nat Neurosci.

[3] Zhu X (2021). Distinct thalamocortical circuits underlie allodynia induced by tissue injury and by depression-like states. Nat Neurosci.

[4] Cao P (2021). Early-life inflammation promotes depressive symptoms in adolescence via microglial engulfment of dendritic spines. Neuron.

[5] Barthas F (2015). The anterior cingulate cortex is a critical hub for pain-induced depression. Biol Psychiatry.

[6] Mayberg HS (1999). Reciprocal limbic-cortical function and negative mood: converging PET findings in depression and normal sadness. Am J Psychiatry.

[7] Drevets WC (2002). Functional anatomical correlates of antidepressant drug treatment assessed using PET measures of regional glucose metabolism. Eur Neuropsychopharmacol.

[8] Koga K (2015). Coexistence of two forms of LTP in ACC provides a synaptic mechanism for the interactions between anxiety and chronic pain. Neuron.

[9] Sellmeijer J (2018). ¬reas 24a/24b ¬sequences. J Neurosci.

[10] Wang J (2011). A single subanesthetic dose of ketamine relieves depression-like behaviors induced by neuropathic pain in rats. Anesthesiology.

[11] Humo M (2020). Ketamine induces rapid and sustained antidepressant-like effects in chronic pain induced depression: Role of MAPK signaling pathway. Prog Neuropsychopharmacol Biol Psychiatry.

[12] Tolias KF (2011). Control of synapse development and plasticity by Rho GTPase regulatory proteins. Prog Neurobiol.

[13] Tolias KF (2005). The Rac1-GEF Tiam1 couples the NMDA receptor to the activity-dependent development of dendritic arbors and spines. Neuron.

[14] Tolias KF (2007). The Rac1 guanine nucleotide exchange factor Tiam1 mediates EphB receptor-dependent dendritic spine development. Proc Natl Acad Sci U S A.

[15] Cheng J (2021). The Rac-GEF Tiam1 promotes dendrite and synapse stabilization of dentate granule cells and restricts hippocampal-dependent memory functions. J Neurosci.

[16] Saneyoshi T (2019). Reciprocal activation within a kinase-effector complex underlying persistence of structural LTP. Neuron.

[17] Loeser JD, Treede RD (2008). The Kyoto protocol of IASP Basic Pain Terminology. Pain.

[18] Decosterd I, Woolf CJ (2000). Spared nerve injury: an animal model of persistent peripheral neuropathic pain. Pain.

[19] Larson AA (1986). Pain threshold changes in adjuvant-induced inflammation: a possible model of chronic pain in the mouse. Pharmacol Biochem Behav.

[20] Arthur WT (2002). XPLN, a guanine nucleotide exchange factor for RhoA and RhoB, but not RhoC. J Biol Chem.

[21] Doan L (2015). Neuroplasticity underlying the comorbidity of pain and depression. Neural Plast.

[22] Price DD (2000). Psychological and neural mechanisms of the affective dimension of pain. Science.

[23] Harte SE (2016). Mechanical conflict system: a novel operant method for the assessment of nociceptive behavior. PLoS One.

[24] Shepherd AJ, Mohapatra DP (2018). Pharmacological validation of voluntary gait and mechanical sensitivity assays associated with inflammatory and neuropathic pain in mice. Neuropharmacology.

[25] Dworkin RH (2007). Pharmacologic management of neuropathic pain: evidence-based recommendations. Pain.

[26] Duman RS (2016). Synaptic plasticity and depression: new insights from stress and rapid-acting antidepressants. Nat Med.

[27] Christoffel DJ (2011). Structural and synaptic plasticity in stress-related disorders. Rev Neurosci.

[28] Bourne JN, Harris KM (2008). Balancing structure and function at hippocampal dendritic spines. Annu Rev Neurosci.

[29] Cingolani LA, Goda Y (2008). Actin in action: the interplay between the actin cytoskeleton and synaptic efficacy. Nat Rev Neurosci.

[30] Kasai H (2003). Structure-stability-function relationships of dendritic spines. Trends Neurosci.

[31] Kopec CD (2006). Glutamate receptor exocytosis and spine enlargement during chemically induced long-term potentiation. J Neurosci.

[32] Lüscher C (2000). Synaptic plasticity and dynamic modulation of the postsynaptic membrane. Nat Neurosci.

[33] Xie Z (2007). Kalirin-7 controls activity-dependent structural and functional plasticity of dendritic spines. Neuron.

[34] Rosenkranz JA (2010). Chronic stress causes amygdala hyperexcitability in rodents. Biol Psychiatry.

[35] Ahmed MR (2015). GRK3 suppresses L-DOPA-induced dyskinesia in the rat model of Parkinson’s disease via its RGS homology domain. Sci Rep.

[36] Chen J (2018). The α2δ-1-NMDA receptor complex is critically involved in neuropathic pain development and gabapentin therapeutic actions. Cell Rep.

[37] Bliss TV, Collingridge GL (1993). A synaptic model of memory: long-term potentiation in the hippocampus. Nature.

[38] Zhuo M (2008). Cortical excitation and chronic pain. Trends Neurosci.

[39] Zhao MG (2005). Roles of NMDA NR2B subtype receptor in prefrontal long-term potentiation and contextual fear memory. Neuron.

[40] Chen T (2014). Adenylyl cyclase subtype 1 is essential for late-phase long term potentiation and spatial propagation of synaptic responses in the anterior cingulate cortex of adult mice. Mol Pain.

[41] Tsvetkov E (2004). Glutamate uptake determines pathway specificity of long-term potentiation in the neural circuitry of fear conditioning. Neuron.

[42] Gao Y (2004). Rational design and characterization of a Rac GTPase-specific small molecule inhibitor. Proc Natl Acad Sci U S A.

[43] Levay M (2013). NSC23766, a widely used inhibitor of Rac1 activation, additionally acts as a competitive antagonist at muscarinic acetylcholine receptors. J Pharmacol Exp Ther.

[44] Drevets WC (2013). Antidepressant effects of the muscarinic cholinergic receptor antagonist scopolamine: a review. Biol Psychiatry.

[46] Correll GE (2004). Subanesthetic ketamine infusion therapy: a retrospective analysis of a novel therapeutic approach to complex regional pain syndrome. Pain Med.

[47] Garcia LSB (2008). Acute administration of ketamine induces antidepressant-like effects in the forced swimming test and increases BDNF levels in the rat hippocampus. Prog Neuropsychopharmacol Biol Psychiatry.

[48] Lai KO, Ip NY (2013). Structural plasticity of dendritic spines: the underlying mechanisms and its dysregulation in brain disorders. Biochim Biophys Acta.

[49] Forrest MP (2018). Dendritic structural plasticity and neuropsychiatric disease. Nat Rev Neurosci.

[50] Woolf CJ (2011). Central sensitization: implications for the diagnosis and treatment of pain. Pain.

[51] Li L (2016). Chloride homeostasis critically regulates synaptic NMDA receptor activity in neuropathic pain. Cell Rep.

[52] Ibarguen-Vargas Y (2008). Multifaceted strain-specific effects in a mouse model of depression and of antidepressant reversal. Psychoneuroendocrinology.

[53] McQuaid RJ (2013). The differential impact of social defeat on mice living in isolation or groups in an enriched environment: plasma corticosterone and monoamine variations. Int J Neuropsychopharmacol.

[54] Ulrich-Lai YM (2006). Limbic and HPA axis function in an animal model of chronic neuropathic pain. Physiol Behav.

[55] Yalcin I (2011). A time-dependent history of mood disorders in a murine model of neuropathic pain. Biol Psychiatry.

[56] Duman RS, Aghajanian GK (2012). Synaptic dysfunction in depression: potential therapeutic targets. Science.

[57] Golden SA (2013). Epigenetic regulation of RAC1 induces synaptic remodeling in stress disorders and depression. Nat Med.

[58] Zhu X S-ketamine exerts antidepressant effects by regulating Rac1 GTPase mediated synaptic plasticity in the hippocampus of stressed rats. Cell Mol Neurobiol.

[59] Martinowich K (2007). New insights into BDNF function in depression and anxiety. Nat Neurosci.

[60] Li N (2010). mTOR-dependent synapse formation underlies the rapid antidepressant effects of NMDA antagonists. Science.

[61] Jiang H (2014). Sensitization of neurons in the central nucleus of the amygdala via the decreased GABAergic inhibition contributes to the development of neuropathic pain-related anxiety-like behaviors in rats. Mol Brain.

[62] Neugebauer V (2004). The amygdala and persistent pain. Neuroscientist.

[63] Neugebauer V (2015). Amygdala pain mechanisms. Handb Exp Pharmacol.

[64] Neugebauer V (2007). The amygdala: different pains, different mechanisms. Pain.

[65] Tye KM (2011). Amygdala circuitry mediating reversible and bidirectional control of anxiety. Nature.

[66] Samineni VK (2021). Cellular, circuit and transcriptional framework for modulation of itch in the central amygdala. Elife.

[67] Ikeda R (2007). NMDA receptor-independent synaptic plasticity in the central amygdala in the rat model of neuropathic pain. Pain.

[68] Gonçalves L (2008). Neuropathic pain is associated with depressive behaviour and induces neuroplasticity in the amygdala of the rat. Exp Neurol.

[69] Manglik A (2016). Structure-based discovery of opioid analgesics with reduced side effects. Nature.

[70] Corder G (2017). Loss of μ opioid receptor signaling in nociceptors, but not microglia, abrogates morphine tolerance without disrupting analgesia. Nat Med.

[71] Corder G (2019). An amygdalar neural ensemble that encodes the unpleasantness of pain. Science.

[72] Fox RJ, Sorenson CA (1994). Bilateral lesions of the amygdala attenuate analgesia induced by diverse environmental challenges. Brain Res.

[73] Woolf CJ (1984). Long term alterations in the excitability of the flexion reflex produced by peripheral tissue injury in the chronic decerebrate rat. Pain.

[74] Rescorla RA (1968). Pavlovian conditioned fear in Sidman avoidance learning. J Comp Physiol Psychol.

[75] Blanchard RJ, Blanchard DC (1969). Passive and active reactions to fear-eliciting stimuli. J Comp Physiol Psychol.

[76] Bolles RC (1970). Species-specific defense reactions and avoidance learning. Psychol Rev.

[77] Chaplan SR (1994). Quantitative assessment of tactile allodynia in the rat paw. J Neurosci Methods.

[78] García-Mata R (2006). Analysis of activated GAPs and GEFs in cell lysates. Methods Enzymol.

[79] Zeng LH (2007). Kainate seizures cause acute dendritic injury and actin depolymerization in vivo. J Neurosci.

[80] Huang W (2013). mTORC2 controls actin polymerization required for consolidation of long-term memory. Nat Neurosci.

[81] Tu YK (2018). The adhesion-GPCR BAI1 promotes excitatory synaptogenesis by coordinating bidirectional trans-synaptic signaling. J Neurosci.

[82] Scala F (2021). Phenotypic variation of transcriptomic cell types in mouse motor cortex. Nature.

[83] Li DP (2008). Pre- and postsynaptic plasticity underlying augmented glutamatergic inputs to hypothalamic presympathetic neurons in spontaneously hypertensive rats. J Physiol.

