# Supplementary material for: TIAM1-mediated synaptic plasticity underlies comorbid depression–like and ketamine antidepressant–like actions in chronic pain
Source: J Clin Invest. 2022 Dec 15;132(24):e158545. doi: 10.1172/JCI158545 (PMC9753999; doi:10.1172/JCI158545)
Supplement: Supplemental data [file jci-132-158545-s111.pdf]

Supplemental materials for

Tiam1-mediated synaptic plasticity underlies comorbid depression-like and ketamine antidepressant-like actions in chronic pain

Qin Ru[1,2], Yungang Lu[1], Ali Bin Saifullah[1], Francisco A Blanco[1,3], Changqun Yao[1], Juan P Cata[4], De-Pei Li[5], Kimberley F Tolias[1,6]*, and Lingyong Li[1,7]*

Corresponding authors: lingyongli@uabmc.edu (L.L.); tolias@bcm.edu (K.F.T.)

This PDF file includes:

Supplemental Figures 1 to 8

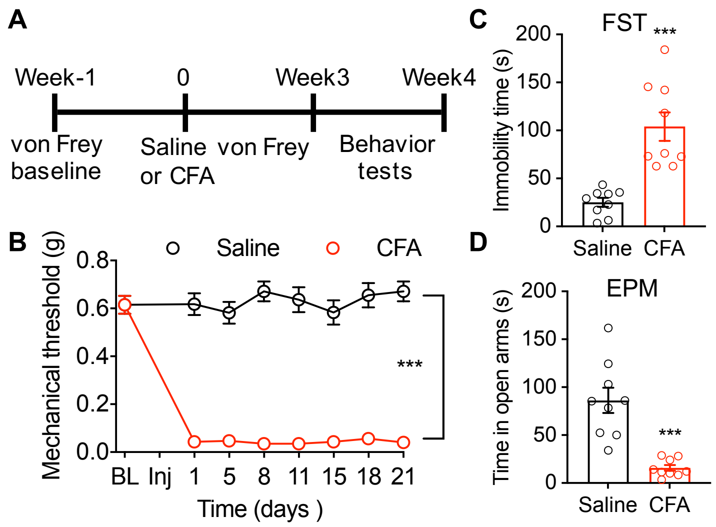

Supplemental Figure 1. Depressive/anxiety-like behaviors are induced by chronic inflammatory pain. (**A**) Experimental paradigm. (**B**) Time course of complete Freund's adjuvant (CFA)-induced sensory pain (n = 9 mice for each group). BL, baseline; Inj, saline or CFA injection. (**C** and **D**) Forced swim test (FST) and elevated plus maze (EPM) show that CFA treatment induces depressive/anxiety-like behaviors 3 weeks after injection (n = 9 mice for each group). Data present means ± s.e.m. ***$P < 0.001$. Two-way ANOVA followed Tukey's *post-hoc* test (B), two-tailed unpaired Student's *t*-test (C,D).

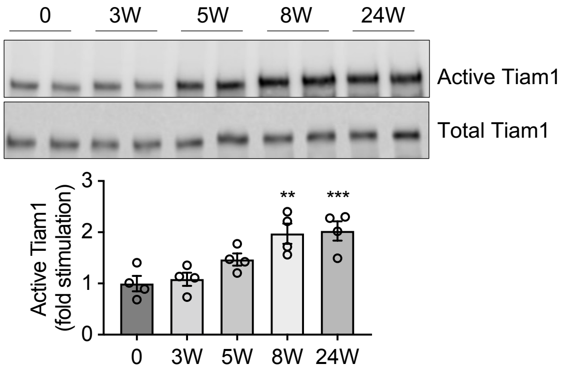

Supplemental Figure 2. The time course of Tiam1 activity in the ACC during the development of comorbid depressive-like symptoms in chronic pain mice. Representative blot and quantification of an affinity-precipitation active GEF assay with GST-Rac1G15A showing Tiam1 activation in the ACC of mice 0, 3, 5, 8, and 24 weeks after spared nerve injury (n = 4 mice). Data represent means ± s.e.m. ** $P < 0.01$, ***$P < 0.001$. One-way ANOVA followed Tukey's *post-hoc* test.

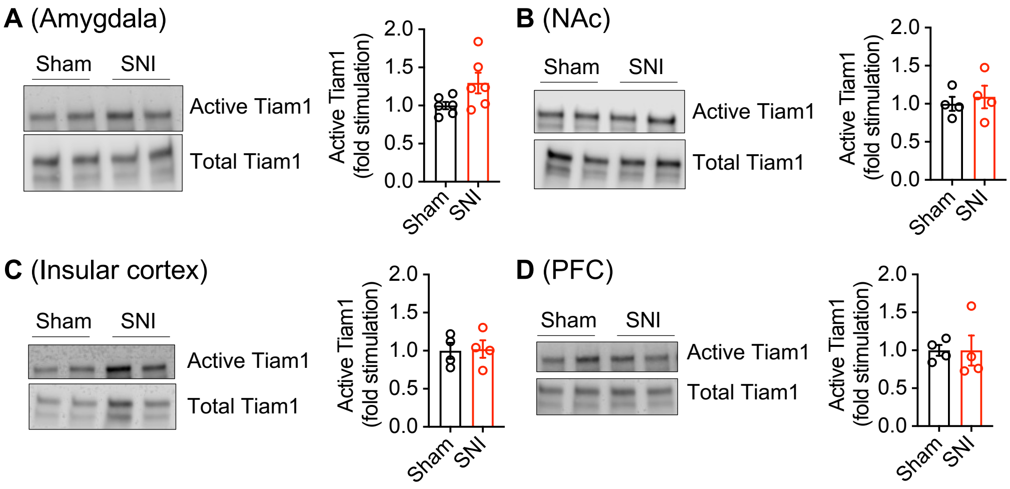

Supplemental Figure 3. Tiam1 activity is not significantly affected in other brain regions of chronic pain mice displaying depressive-like behaviors. Representative blots and quantification of an affinity-precipitation active GEF assay with GST-Rac1G15A showing Tiam1 activity in the amygdala (**A**), nucleus accumbens (NAc) (**B**), insular cortex (**C**), and prefrontal cortex (PFC) (**D**) 7 weeks after sham or spared nerve injury (SNI) (n = 4 mice). No significant difference was detected between sham and SNI groups. Data represent means ± s.e.m. Two-tailed unpaired Student's *t*-test (A-D).

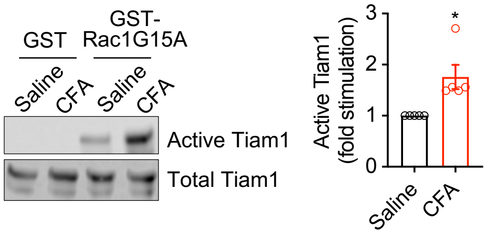

Supplemental Figure 4. Chronic inflammatory pain activates Tiam1 in the ACC. Active Tiam1 was detected by an affinity-precipitation assay using GST-Rac1G15A, which preferentially binds to activated GEFs. Tiam1 was precipitated from lysates prepared from the ACC of mice 3 weeks after saline or CFA injection. Total Tiam1 levels are also shown. (n = 5 mice for each group). Data present means ± s.e.m. * $P < 0.05$. Two-tailed unpaired Student's *t*-test.

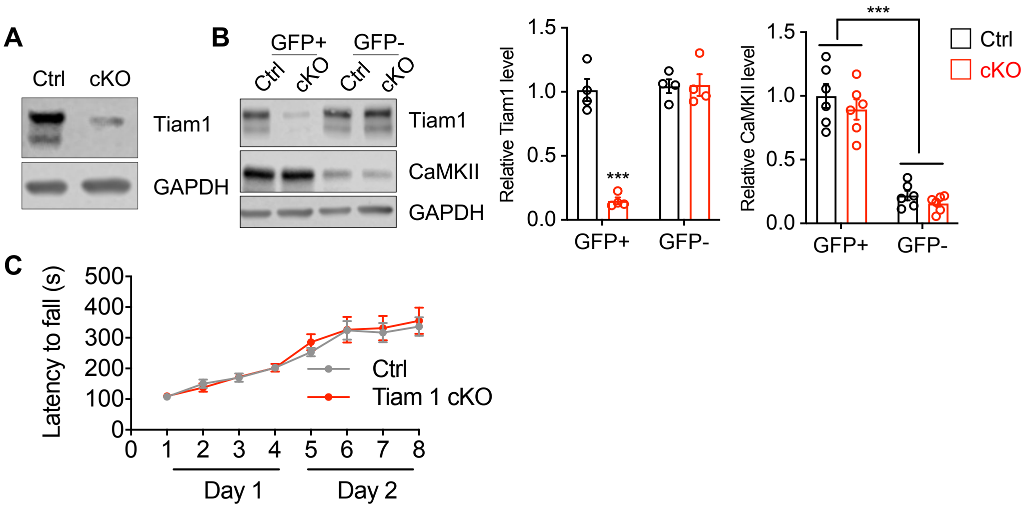

Supplemental Figure 5. Tiam1 cKO mice. (**A**) Representative immunoblots of forebrain lysate from control littermates (Ctrl) and Tiam1 cKO (CaMKIIα-Cre::Tiam1$^{flox/flox}$) mice were probed with antibodies against Tiam1 and GAPDH. (**B**) Selective deletion of Tiam1 from CaMKIIα neurons in Tiam1 cKO mice. Cre-dependent AAV vector expressing eGFP (AAV8-pCAG-DIO-eGFP) was injected into the ACC of the control mice (CaMKIIα-Cre) and Tiam1 cKO mice (CaMKIIα-Cre::Tiam1$^{flox/flox}$). eGFP-expressing ACC neurons were isolated with flow cytometry. Representative blot and quantification indicated selective deletion of Tiam1 from CaMKIIα neurons in Tiam1 cKO mice (n = 4 mice for each group). (**C**) Control (Ctrl) and Tiam1 cKO mice were tested on an accelerating rotarod for two days (4 trials per day), and their motor performance was compared. No significant difference was detected between the two groups of mice (Ctrl, n = 12 mice; cKO n = 14 mice). Data represent means ± s.e.m. ***$P < 0.001$. Two-way ANOVA followed Tukey's *post-hoc* test (C,D).

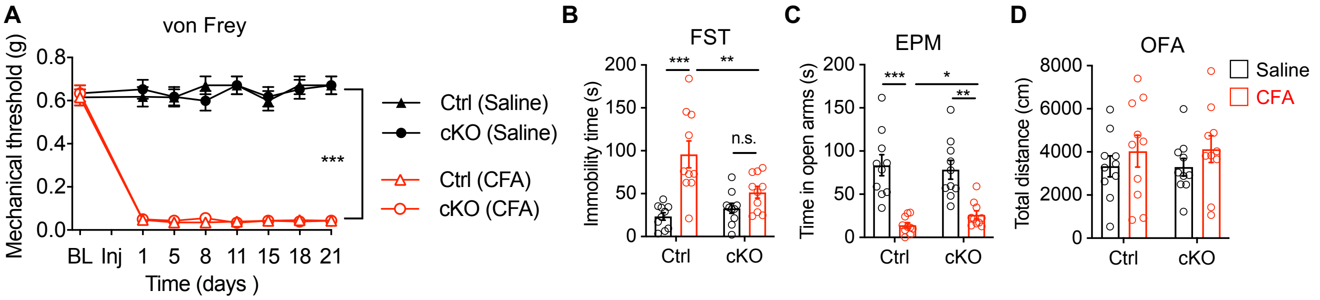

Supplemental Figure 6. Genetic deletion of Tiam1 from postnatal forebrain excitatory neurons reduces inflammatory pain-induced depressive/anxiety-like behaviors. (**A**) Time course of CFA-induced sensory pain showed that Tiam1 cKO mice displayed no difference in mechanical allodynia compared to controls (Ctrl) before or during the 3 weeks following saline or CFA injection (n = 10 mice for each group). BL, baseline. Inj, saline or CFA injection. (**B** and **C**) Behavioral tests demonstrated that Tiam1 cKO mice showed reduced inflammatory pain-induced depressive/anxiety-like behaviors in FST and EPM tests compared to control mice (Ctrl). (**D**) Open field activity test showed that chronic neuropathic pain did not affect locomotion in control or Tiam1 cKO mice. n = 10 mice for each group in B,C,D. Data present means ± s.e.m. * $P < 0.05$, ** $P < 0.01$, ***$P < 0.001$, n.s., no significance. Two-way ANOVA followed Tukey's *post-hoc* test.

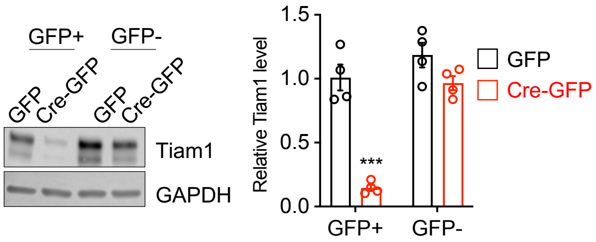

Supplemental Figure 7. The selective deletion of Tiam1 in AAV-Cre-GFP transduced ACC neurons in Tiam1 floxed mice. GFP-positive ACC neurons were isolated with flow cytometry from AAV-GFP or AAV-Cre-GFP injected ACC neurons in Tiam1 floxed mice. Representative blot and quantification indicated selective deletion of Tiam1 from AAV-Cre-GFP transduced ACC neurons in Tiam1 floxed mice (n = 4 mice for each group). Data represent means ± s.e.m. ***$P < 0.001$. Two-way ANOVA followed Tukey's *post-hoc* test.

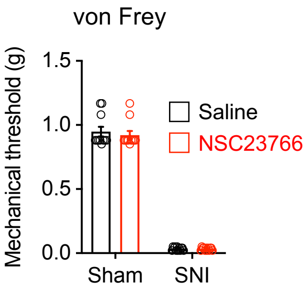

Supplemental Figure 8. NSC23766 treatment (1 mg/kg) does not affect SNI-induced pain hypersensitivity. Low dose administration of NSC23766 (1 mg/kg, i.p.) had no effect on SNI-induced pain hypersensitivity (7 weeks after SNI surgery). Data presented as means ± s.e.m. (n = 11 mice for each group). Two-way ANOVA followed Tukey's *post-hoc* test.
